# Supplementary material for: Genotyping structural variants in pangenome graphs using the vg toolkit
Source: Genome Biol. 2020 Feb 12;21:35. doi: 10.1186/s13059-020-1941-7 (PMC7017486; doi:10.1186/s13059-020-1941-7)
Supplement: Supplementary file 1 — Table S1-S6, Figure S1-S18, Supplementary information. [file 13059_2020_1941_MOESM1_ESM.pdf]

Genotyping structural variants in pangenome graphs using the vg toolkit

Authors

Glenn Hickey[1,☯] , David Heller[1,2,☯] , Jean Monlong[1,☯] , Jonas A. Sibbesen[1] , Jouni Sirén[1] , Jordan Eizenga[1] , Eric T. Dawson[3,4] , Erik Garrison[1] , Adam M. Novak[1] , Benedict Paten[1,†]

☯ — These authors contributed equally to this work

† — To whom correspondence should be addressed: bpaten@ucsc.edu

1. UC Santa Cruz Genomics Institute, University of California, Santa Cruz, California, USA
2. Max Planck Institute for Molecular Genetics, Berlin, Germany
3. Department of Genetics, University of Cambridge, Cambridge, UK
4. Division of Cancer Epidemiology and Genetics, National Cancer Institute, Rockville, Maryland, USA

Supplementary Material

- Table S1: Genotyping evaluation on the HGSVC dataset.
- Table S2: Genotyping evaluation on the Genome in a Bottle dataset.
- Table S3: Genotyping evaluation on the pseudo-diploid genome built from CHM cell lines in Audano et al.(1).
- Table S4): Calling evaluation on the SVPOP dataset.
- Table S5: Calling evaluation on the SVPOP dataset in different sets of regions for the HG5014 individual.
- Table S6: Breakpoint fine-tuning using graph augmentation from the read alignment.
- Table S7: Compute resources required for analysis of sample HG00514 on the HGSVC dataset.

- Fig. S1 Genotyping evaluation on the HGSVC dataset using simulated reads.
- Fig. S2 Calling evaluation on the HGSVC dataset using simulated reads.
- Fig. S3: Genotyping evaluation on the HGSVC dataset using real reads.
- Fig. S4: Calling evaluation on the HGSVC dataset using real reads.
- Fig. S5: Average number of genotyped variants overlapping one variant from the truth set.
- Fig. S6: Evaluation across different repeat profiles.
- Fig. S7: Genotyping evaluation on the Genome in a Bottle dataset.
- Fig. S8: Calling evaluation on the Genome in a Bottle dataset.
- Fig. S9: Genotyping evaluation on the CHM pseudo-diploid dataset.
- Fig. S10: Calling evaluation on the CHM pseudo-diploid dataset.
- Fig. S11: Calling evaluation on the SVPOP dataset.
- Fig. S12: Evaluation across different sets of regions in HG00514 (SVPOP dataset).
- Fig. S13: Mapping comparison on graphs of the *five strains set*.
- Fig. S14: Mapping comparison on graphs of the *all strains set*.
- Fig. S15: SV genotyping comparison using all reads.
- Fig. S16: Breakpoint fine-tuning using augmentation through "vg call".
- Fig. S17: Overview of the SV evaluation by the *sveval* package.
- Fig. S18: Benchmark summary when using a more stringent matching criterion.

- Supplementary Information

Supplementary Tables

Table S1: Genotyping evaluation on the HGSVC dataset. Precision, recall and F1 score for the call set with the best F1 score. The best F1 scores were achieved with no filtering in the vast majority of cases (see Fig. S1 and S3). The numbers in parentheses corresponds to the results in non-repeat regions.

Experiment	Method	Type	Precision	Recall	F1
Simulated reads	vg	INS	0.863 (0.918)	0.841 (0.911)	0.852 (0.914)
		DEL	0.85 (0.961)	0.796 (0.959)	0.822 (0.96)
	Paragraph	INS	0.581 (0.831)	0.749 (0.804)	0.654 (0.818)
		DEL	0.707 (0.853)	0.73 (0.811)	0.718 (0.832)
	BayesTyper	INS	0.915 (0.944)	0.839 (0.907)	0.876 (0.925)
		DEL	0.894 (0.983)	0.804 (0.932)	0.847 (0.957)
	SVTyper	DEL	0.811 (0.844)	0.328 (0.74)	0.467 (0.788)
	Delly Genotyper	INS	0.757 (0.857)	0.094 (0.225)	0.167 (0.356)
		DEL	0.681 (0.88)	0.684 (0.823)	0.682 (0.851)
Real reads	vg	INS	0.5 (0.714)	0.492 (0.712)	0.496 (0.713)
		DEL	0.629 (0.864)	0.519 (0.787)	0.569 (0.824)
	Paragraph	INS	0.404 (0.638)	0.555 (0.595)	0.468 (0.616)
		DEL	0.595 (0.787)	0.554 (0.659)	0.574 (0.717)
	BayesTyper	INS	0.599 (0.757)	0.253 (0.436)	0.356 (0.553)
		DEL	0.625 (0.909)	0.324 (0.471)	0.427 (0.62)
	SVTyper	DEL	0.69 (0.728)	0.242 (0.59)	0.358 (0.652)
	Delly Genotyper	INS	0.524 (0.632)	0.068 (0.175)	0.12 (0.274)
		DEL	0.556 (0.834)	0.429 (0.596)	0.484 (0.695)

Table S2: Genotyping evaluation on the Genome in a Bottle dataset. Precision, recall and F1 score for the call set with the best F1 score. The best F1 scores were achieved with no filtering in the vast majority of cases (see Fig. S7). The numbers in parentheses corresponds to the results in non-repeat regions.

Method	Type	Precision	Recall	F1
vg	INS	0.649 (0.776)	0.618 (0.73)	0.633 (0.752)
	DEL	0.696 (0.807)	0.691 (0.795)	0.694 (0.801)
Paragraph	INS	0.699 (0.827)	0.673 (0.768)	0.686 (0.796)
	DEL	0.75 (0.9)	0.726 (0.815)	0.737 (0.855)
BayesTyper	INS	0.777 (0.879)	0.285 (0.379)	0.417 (0.53)
	DEL	0.807 (0.884)	0.514 (0.694)	0.628 (0.778)
SVTyper	DEL	0.743 (0.817)	0.341 (0.496)	0.467 (0.618)
Delly Genotyper	INS	0.804 (0.888)	0.178 (0.269)	0.292 (0.413)
	DEL	0.721 (0.821)	0.644 (0.766)	0.68 (0.793)

Table S3: Genotyping evaluation on the pseudo-diploid genome built from CHM cell lines in Audano et al.(1). The numbers in parentheses corresponds to the results in non-repeat regions.

Method	Type	Precision	Recall	F1
vg	INS	0.783 (0.907)	0.773 (0.895)	0.778 (0.901)
	DEL	0.787 (0.962)	0.635 (0.901)	0.703 (0.93)
SMRT-SV v2 Genotyper	INS	0.819 (0.934)	0.582 (0.712)	0.681 (0.808)
	DEL	0.848 (0.973)	0.63 (0.839)	0.723 (0.901)

Table S4: Calling evaluation on the SVPOP dataset. Combined results for the HG00514, HG00733 and NA19240 individuals, 3 of the 15 individuals used to generate the high-quality SV catalog in Audano et al.(1).

**Method**	**Region**	**Type**	**TP**	**FP**	**FN**	**Precision**	**Recall**	**F1**
vg	all	INS	23430	18414	18181	0.564	0.563	0.564
		DEL	14717	7033	15254	0.677	0.491	0.569
		INV	41	16	159	0.719	0.205	0.319
	non-repeat	INS	8078	3303	1761	0.709	0.821	0.761
		DEL	6585	1033	1040	0.862	0.864	0.863
		INV	37	15	90	0.712	0.291	0.413
Paragraph	all	INS	24342	25618	17269	0.493	0.585	0.535
		DEL	16986	13376	12985	0.571	0.567	0.569
		INV	47	24	153	0.662	0.235	0.347
	non-repeat	INS	7843	3270	1996	0.706	0.797	0.749
		DEL	6523	1000	1102	0.866	0.856	0.860
		INV	39	12	88	0.765	0.307	0.438
SMRT-SV v2 Genotyper	all	INS	16297	26006	25314	0.397	0.392	0.394
		DEL	11797	10054	18174	0.544	0.394	0.457
	non-repeat	INS	4475	4645	5364	0.493	0.455	0.473
		DEL	4986	1322	2639	0.788	0.654	0.715

Table S5: Calling evaluation on the SVPOP dataset in different sets of regions for the HG5014 individual.

Method	Region	Type	TP	FP	FN	Precision	Recall	F1
vg	all	INS	7764	6109	6270	0.567	0.553	0.560
		DEL	4841	2260	5066	0.684	0.489	0.570
		INV	16	6	49	0.727	0.246	0.368
	repeat	INS	5091	5150	5766	0.507	0.469	0.487
		DEL	2684	1922	4648	0.590	0.366	0.452
		INV	1	0	9	1.000	0.100	0.182
	non-repeat	INS	2662	979	521	0.732	0.836	0.781
		DEL	2085	322	388	0.865	0.843	0.854
		INV	14	6	26	0.700	0.350	0.467
	called in SMRT-SV v2 Genotyper	INS	3682	4752	1836	0.444	0.667	0.534
		DEL	2769	1779	1356	0.609	0.671	0.639
		INV	16	6	49	0.727	0.246	0.368
	not called in SMRT-SV v2 Genotyper	INS	3867	291	4649	0.931	0.454	0.610
		DEL	1976	102	3797	0.952	0.342	0.503
SMRT-SV v2 Genotyper	all	INS	5254	8562	8780	0.394	0.374	0.384
		DEL	3743	3367	6164	0.535	0.378	0.443
	repeat	INS	3858	7119	6999	0.368	0.355	0.362
		DEL	2141	2906	5191	0.438	0.292	0.350
	non-repeat	INS	1394	1464	1789	0.493	0.438	0.464
		DEL	1550	443	923	0.778	0.627	0.694
	called in SMRT-SV v2 Genotyper	INS	4360	5619	1158	0.445	0.790	0.570
		DEL	3272	2554	853	0.568	0.793	0.662
	not called in SMRT-SV v2 Genotyper	INS	111	101	8405	0.549	0.013	0.025
		DEL	211	50	5562	0.792	0.036	0.070

Table S6: Breakpoint fine-tuning using graph augmentation from the read alignment. For deletions and inversions, either one or both breakpoints were shifted to introduce errors in the input VCF. For insertions, the insertion location and sequence contained errors. In all cases, the errors affected 1-10 bp.

SV type	Error type	Breakpoint	Variant	Proportion	Mean size (bp)	Mean error (bp)
DEL	one end	incorrect	220	0.219	422.655	6.095
		fine-tuned	784	0.781	670.518	5.430
	both ends	incorrect	811	0.814	826.070	6.275
		fine-tuned	185	0.186	586.676	2.232
INS	location/seq	incorrect	123	0.062	428.724	6.667
		fine-tuned	1877	0.938	440.043	6.439
INV	one end	incorrect	868	0.835	762.673	5.161
		fine-tuned	172	0.165	130.244	5.884
	both ends	incorrect	950	0.992	556.274	5.624
		fine-tuned	8	0.008	200.000	1.375

Table S7: Compute resources required for analysis of sample HG00514 on the HGSVC dataset. elly Genotyper, SVTyper and Paragraph start from a set of aligned reads, hence we also show the running time for read alignment with `bwa mem` (2). For BayesTyper, the numbers include both khmer counting with kmc and genotyping. We note that BayesTyper integrated variant calls from GATK haplotypecaller(3) and Platypus(4), derived from reads mapped with bwa mem(2). The numbers shown for BayesTyper does not include this variant discovery pipeline. More information in the supplementary information below.

Tool	Wall Time (m)	Cores	Nodes	Max Memory (G)
**vg**				
vg construction	49	8	1 i3.8xlarge	0.4
xg index	13	8	1 i3.8xlarge	48
snarls index	23	1	50 i3.8xlarge	17
gcsa2 index	792	16	1 i3.8xlarge	45
mapping	177	32	50 r3.8xlarge	32
genotyping (pack + call)	56	10	1 i3.4xlarge	63
**BayesTyper**	90	24	1 i3.8xlarge	36
**bwa mem**	240	32	1 i3.8xlarge	14
**Delly Genotyper**	69	1	1 i3.8xlarge	69
**SVTyper**	477	1	1 i3.8xlarge	0.7
**Paragraph**	76	32	1 i3.8xlarge	5.9

Supplementary Figures

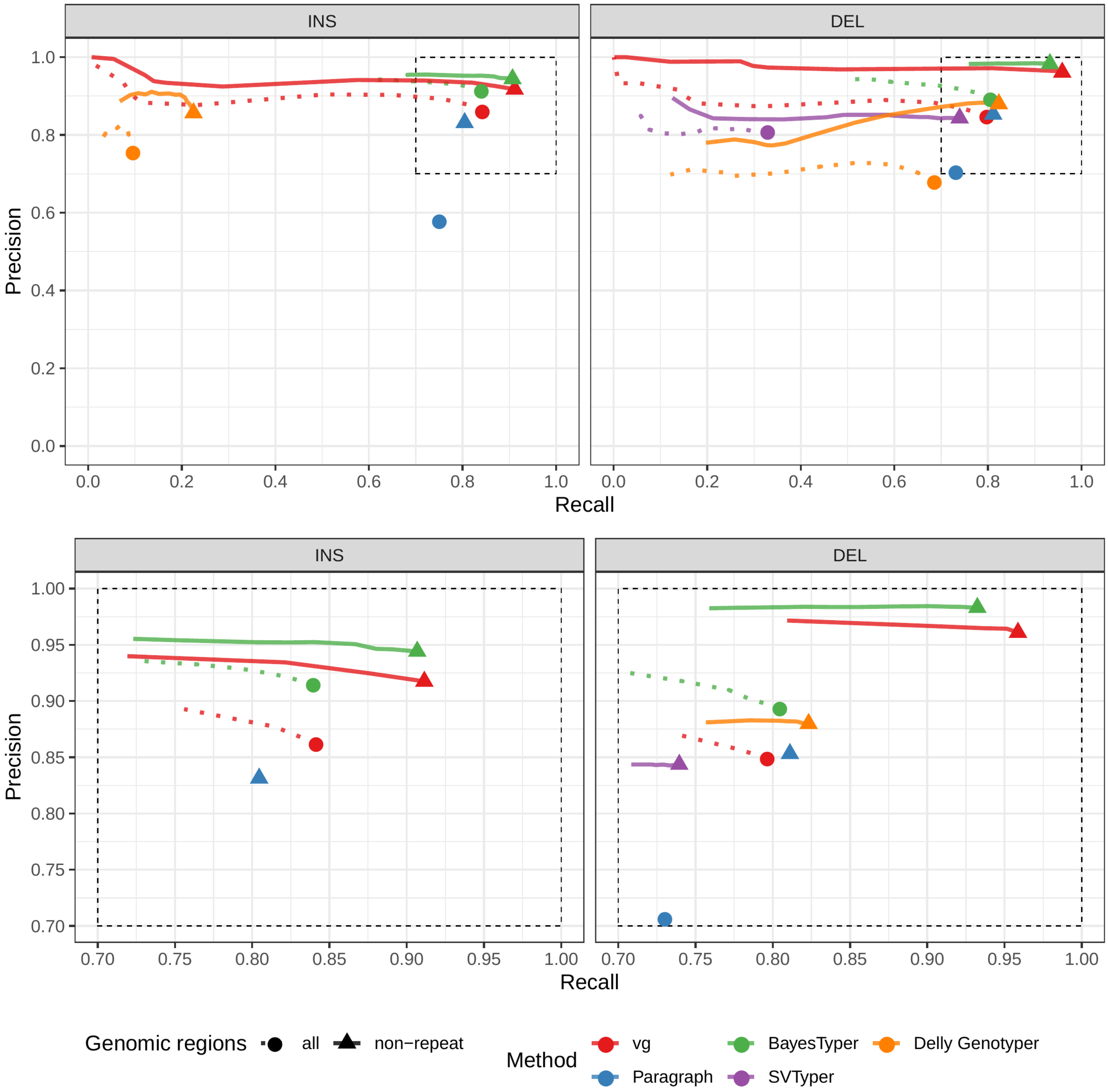

Fig. S1: Genotyping evaluation on the HGSVC dataset using simulated reads. Reads were simulated from the HG00514 individual. The bottom panel zooms on the part highlighted by a dotted rectangle.

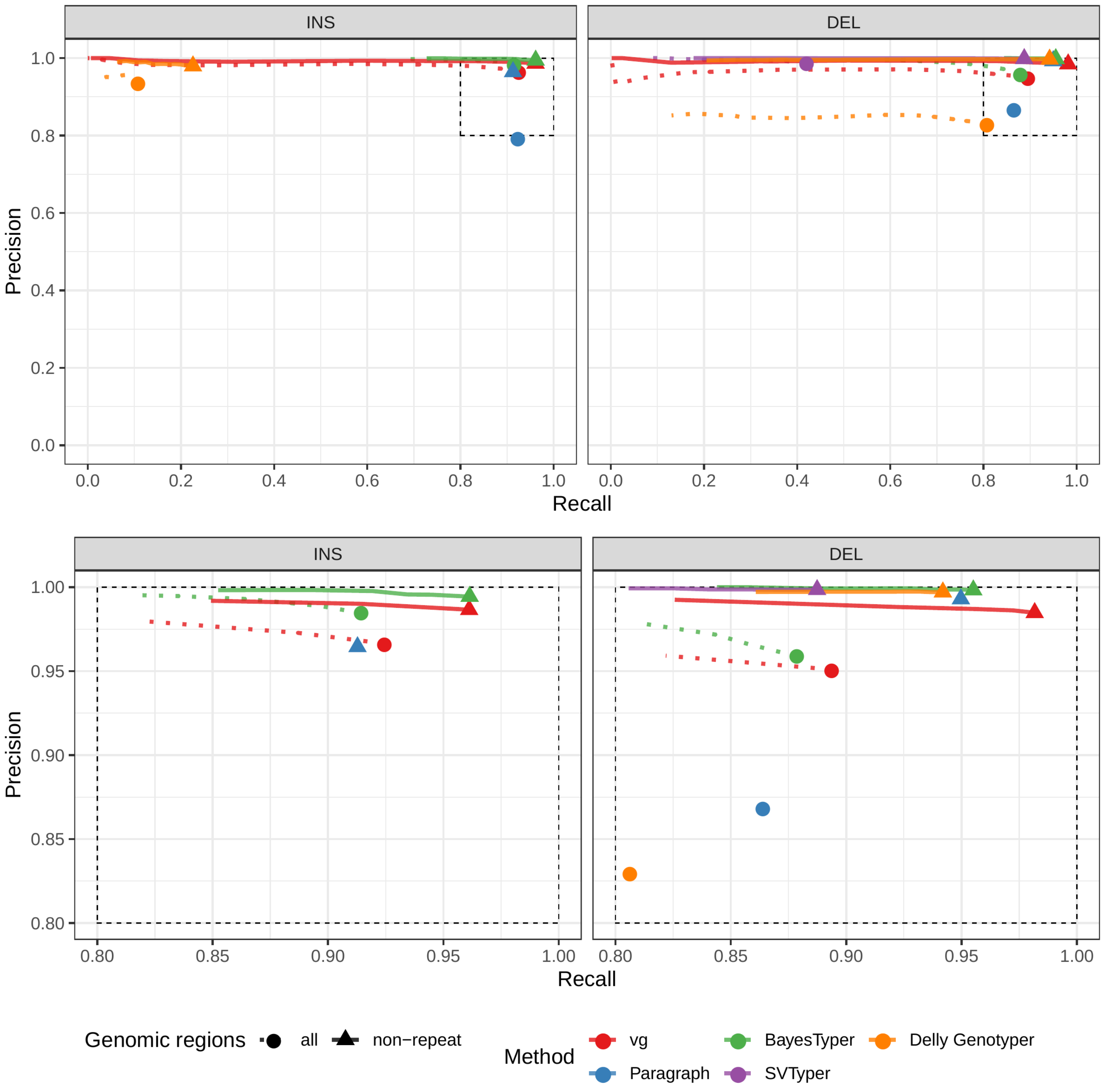

Fig. S2: Calling evaluation on the HGSVC dataset using simulated reads. Reads were simulated from the HG00514 individual. The bottom panel zooms on the part highlighted by a dotted rectangle.

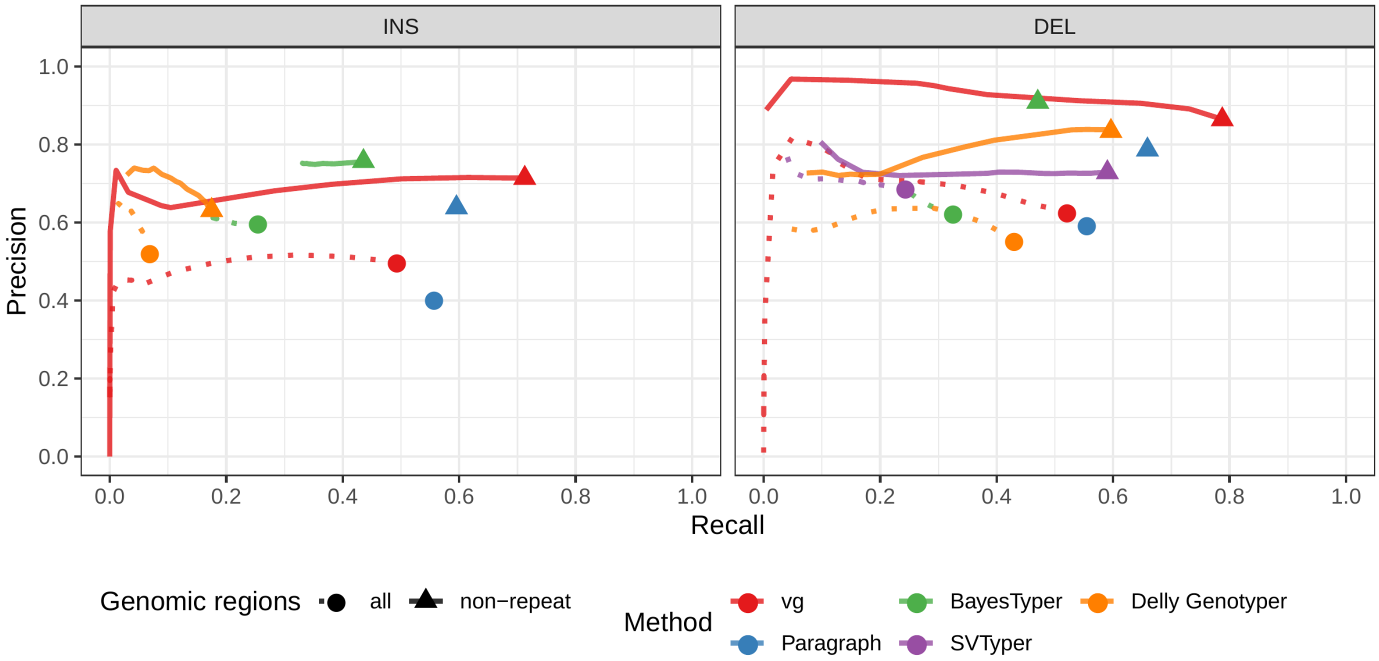

Fig. S3: Genotyping evaluation on the HGSVC dataset using real reads. Combined results across the HG00514, HG00733 and NA19240.

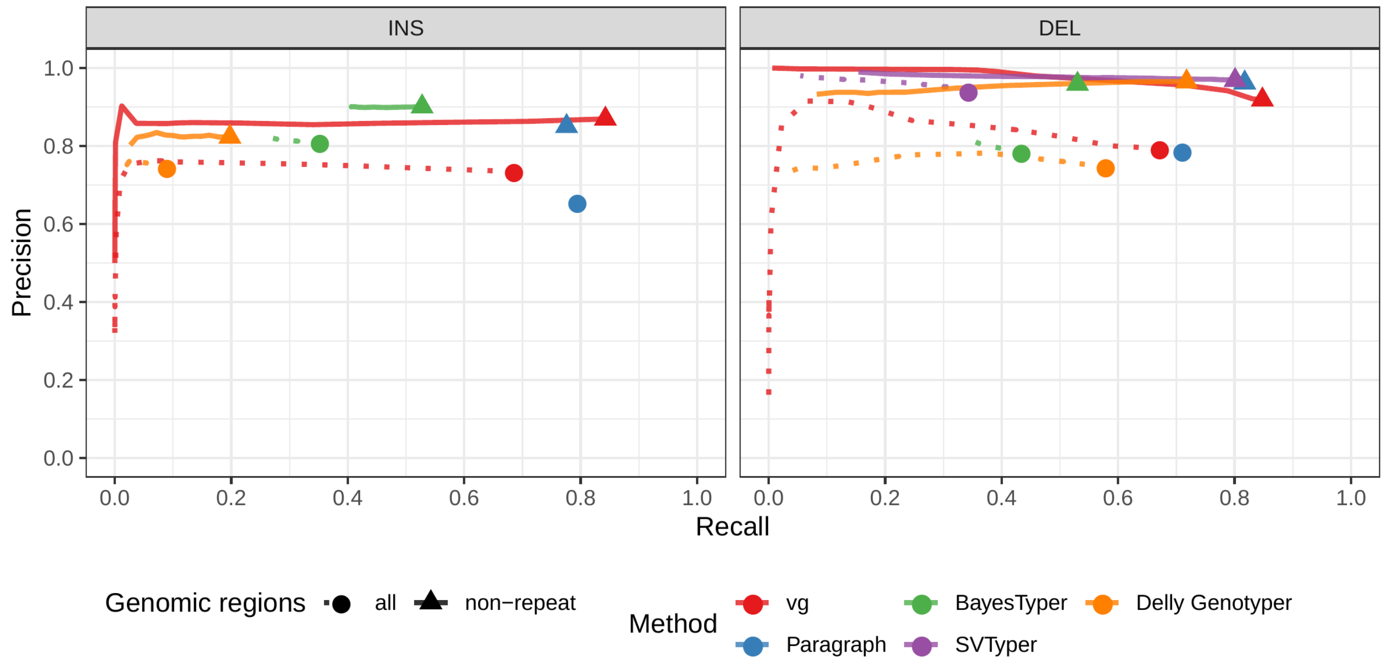

Fig. S4: Calling evaluation on the HGSVC dataset using real reads. Combined results across the HG00514, HG00733 and NA19240.

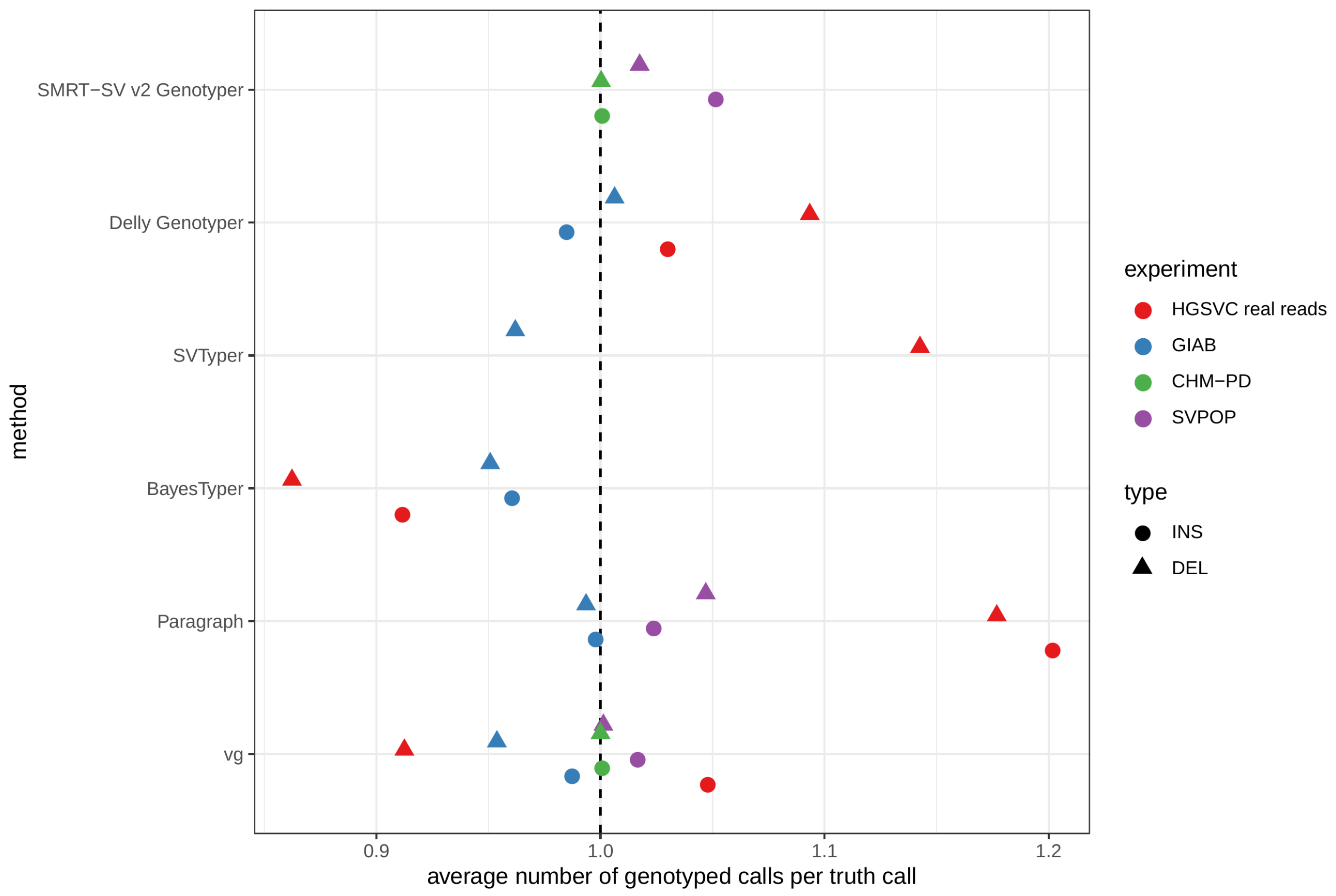

Fig. S5: Average number of genotyped variants overlapping one variant from the truth set. To evaluate the genotyping performance, each genotyped variant is matched to variants in the truth set. A same variant can match to several variant in the other set because of variant fragmentation or when the truth set contains potentially duplicated SVs. This x-axis shows the average number of genotyped variants that were matched per truth-set variant. For example, a value higher than 1 means that variants in the truth were often matched to multiple genotyped variants ("over-genotyping").

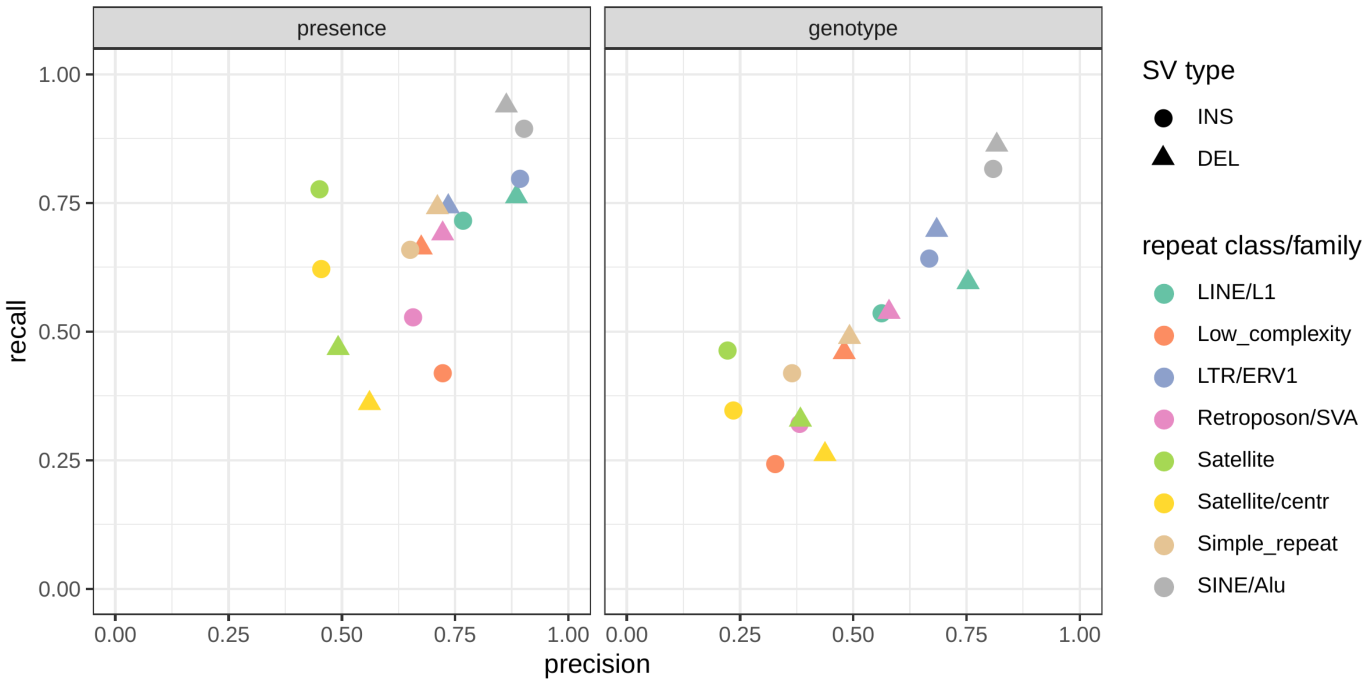

Fig. S6: Evaluation across different repeat profiles. The deleted/inserted sequence was annotated with RepeatMasker (color). The precision and recall was recomputed on each of the most frequent repeat families.

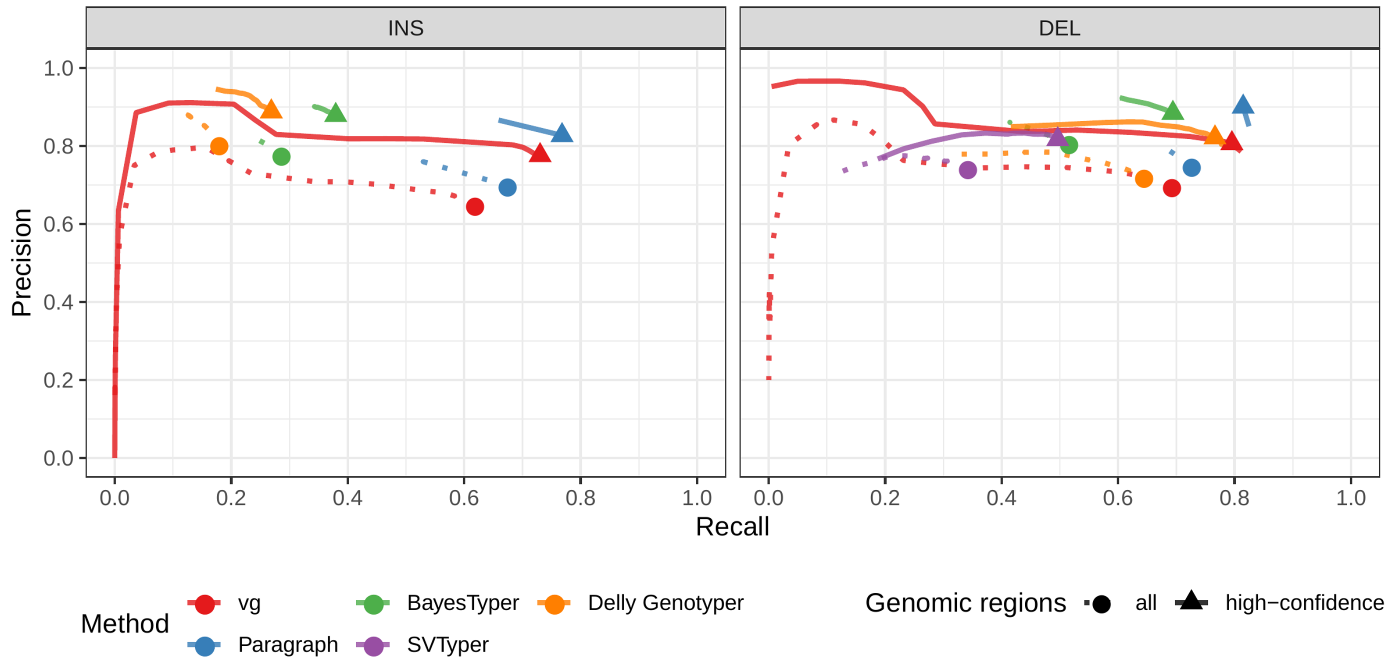

Fig. S7: Genotyping evaluation on the Genome in a Bottle dataset. Predicted genotypes on HG002 were compared to the high-quality SVs from this same individual.

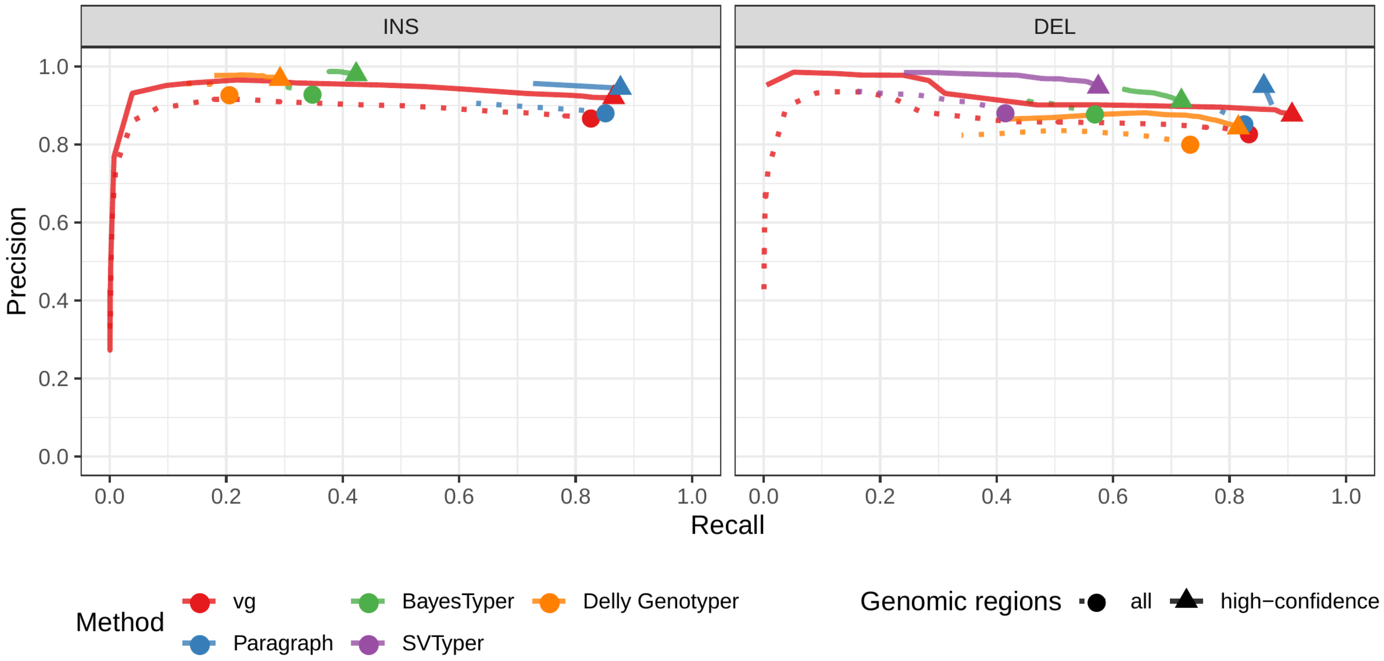

Fig. S8: Calling evaluation on the Genome in a Bottle dataset. Calls on HG002 were compared to the high-quality SVs from this same individual.

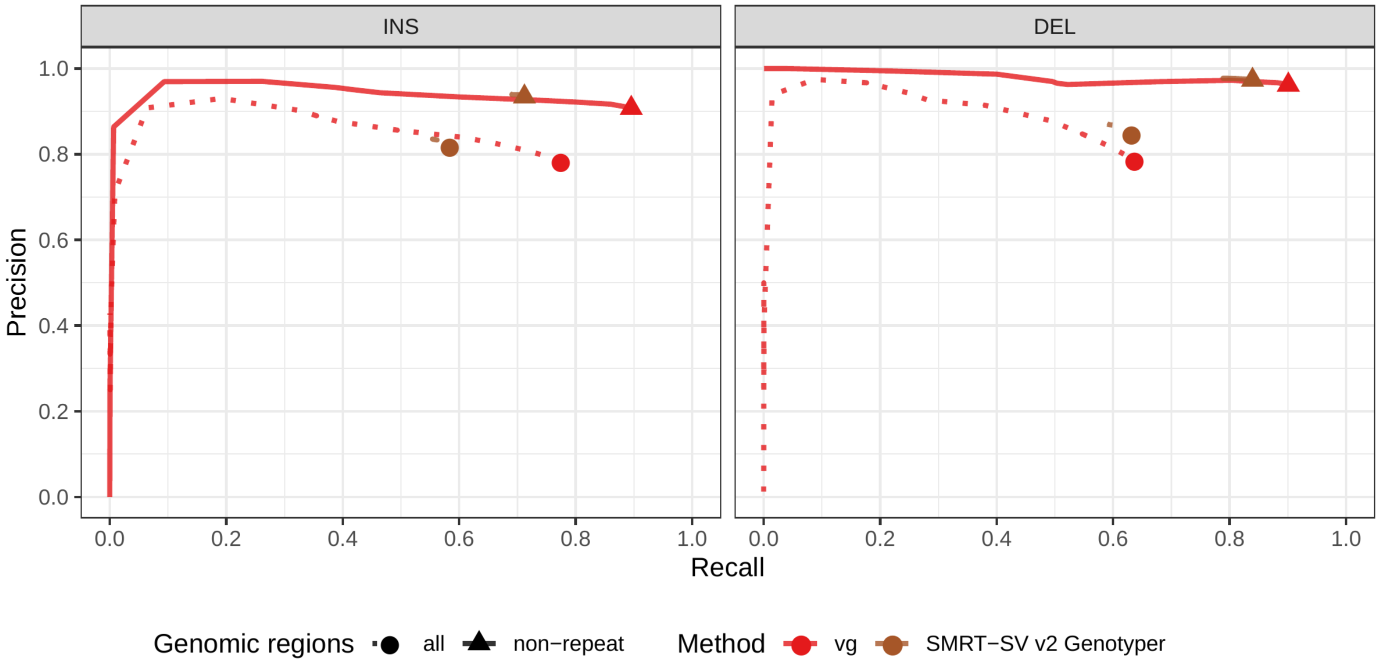

Fig. S9: Genotyping evaluation on the CHM pseudo-diploid dataset. The pseudo-diploid genome was built from CHM cell lines and used to train SMRT-SV v2 Genotyper in Audano et al.(1) The bottom panel zooms on the part highlighted by a dotted rectangle.

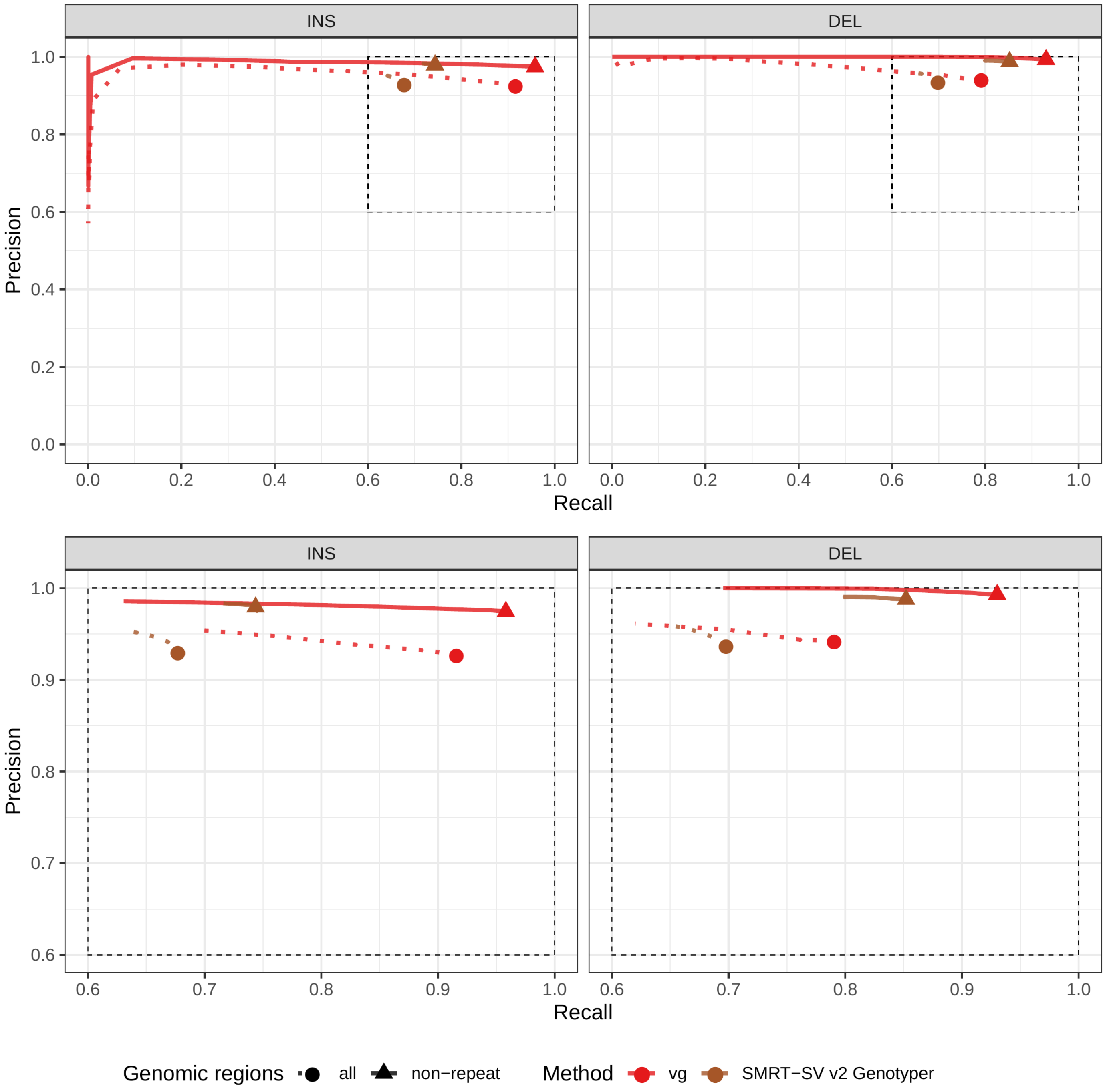

Fig. S10: Calling evaluation on the CHM pseudo-diploid dataset. The pseudo-diploid genome was built from CHM cell lines and used to train SMRT-SV v2 Genotyper in Audano et al.(1)

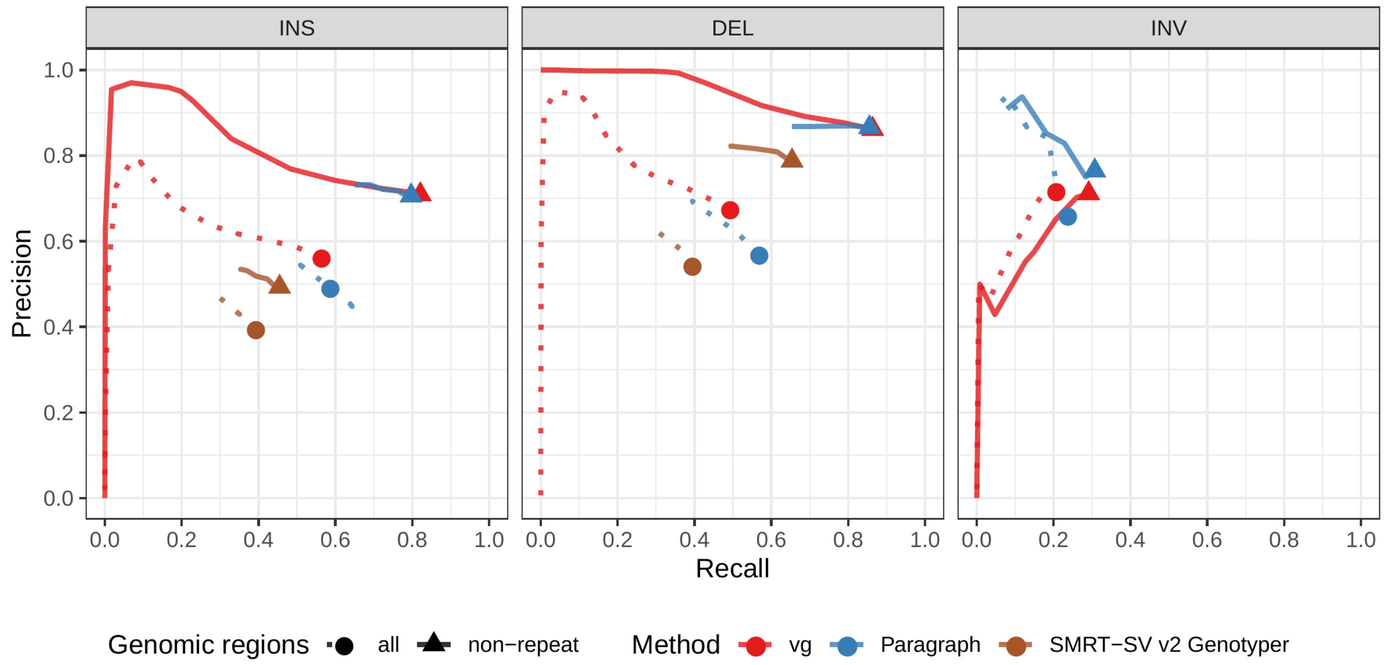

Fig. S11: Calling evaluation on the SVPOP dataset. Combined results across the HG00514, HG00733 and NA19240.

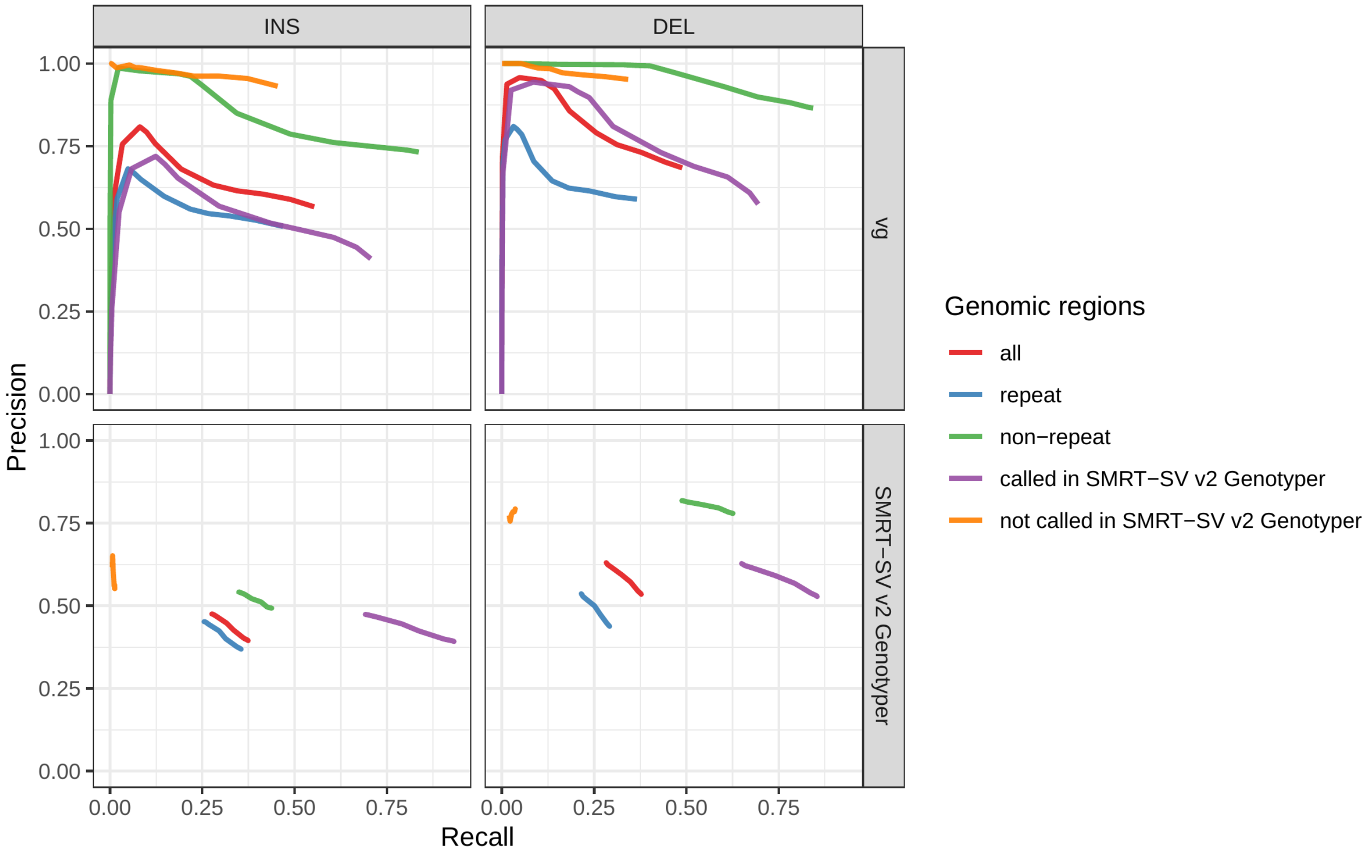

Fig. S12: Evaluation across different sets of regions in HG00514 (SVPOP dataset). Calling evaluation.

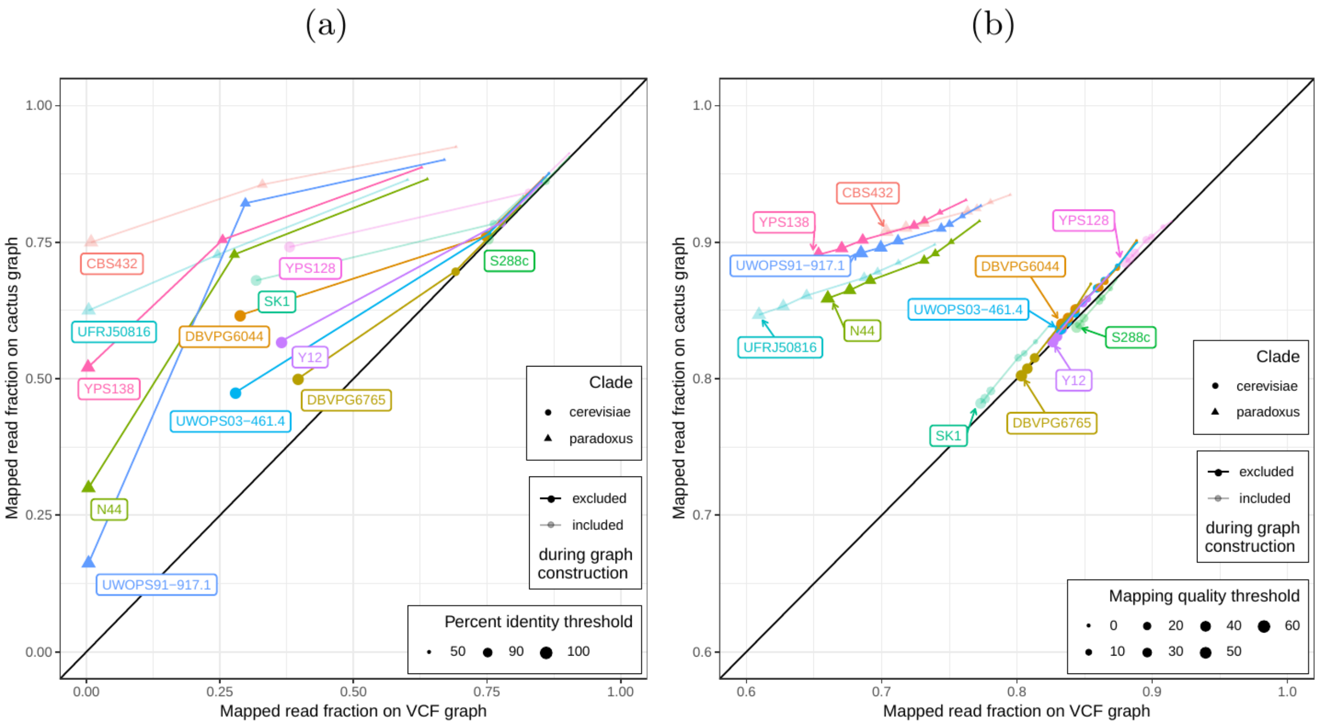

Fig. S13: Mapping comparison on graphs of the *five strains set*. Short reads from all 12 yeast strains were aligned to both graphs. The fraction of reads mapped to the cactus graph (y-axis) and the VCF graph (x-axis) are compared. a) Stratified by percent identity threshold. b) Stratified by mapping quality threshold. Colors and shapes represent the 12 strains and two clades, respectively. Transparency indicates whether the strain was included or excluded in the graphs.

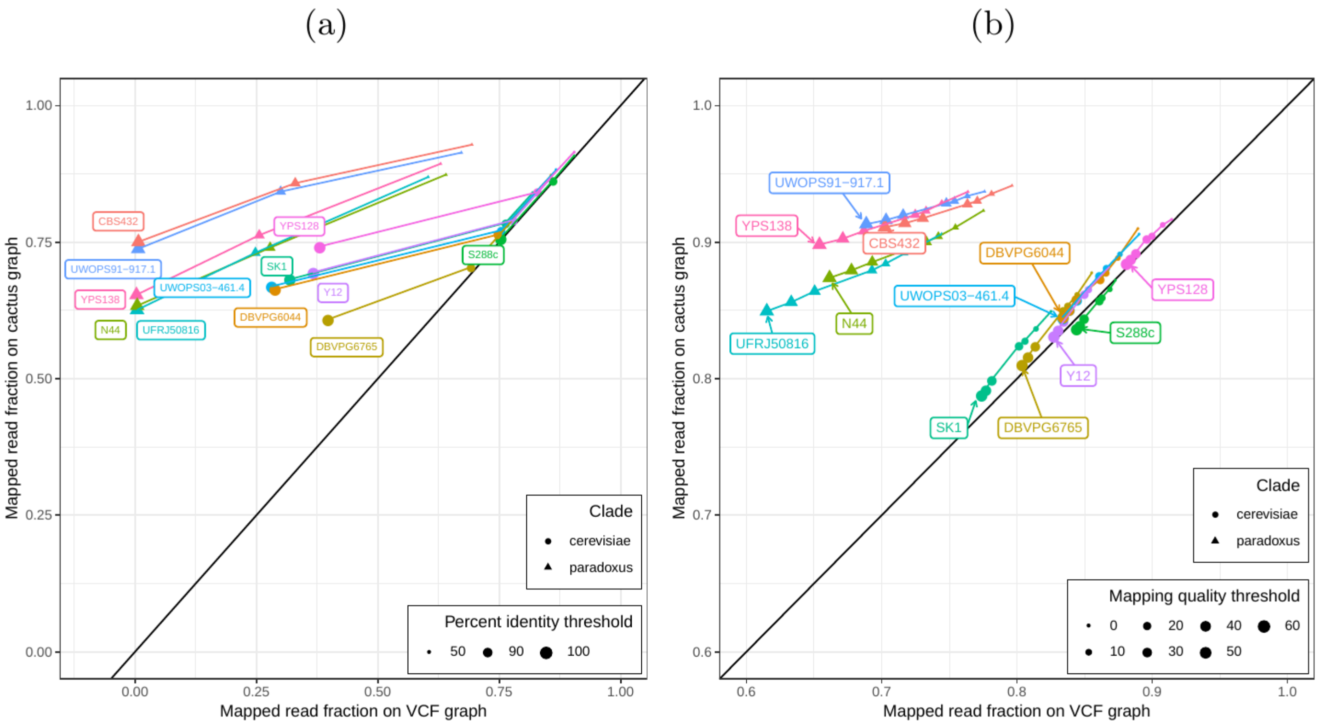

Fig. S14: Mapping comparison on graphs of the *all strains set.* Short reads from all 12 yeast strains were aligned to both graphs. The fraction of reads mapped to the *cactus graph* (y-axis) and the *VCF graph* (x-axis) are compared. a) Stratified by percent identity threshold. b) Stratified by mapping quality threshold. Colors and shapes represent the 12 strains and two clades, respectively.

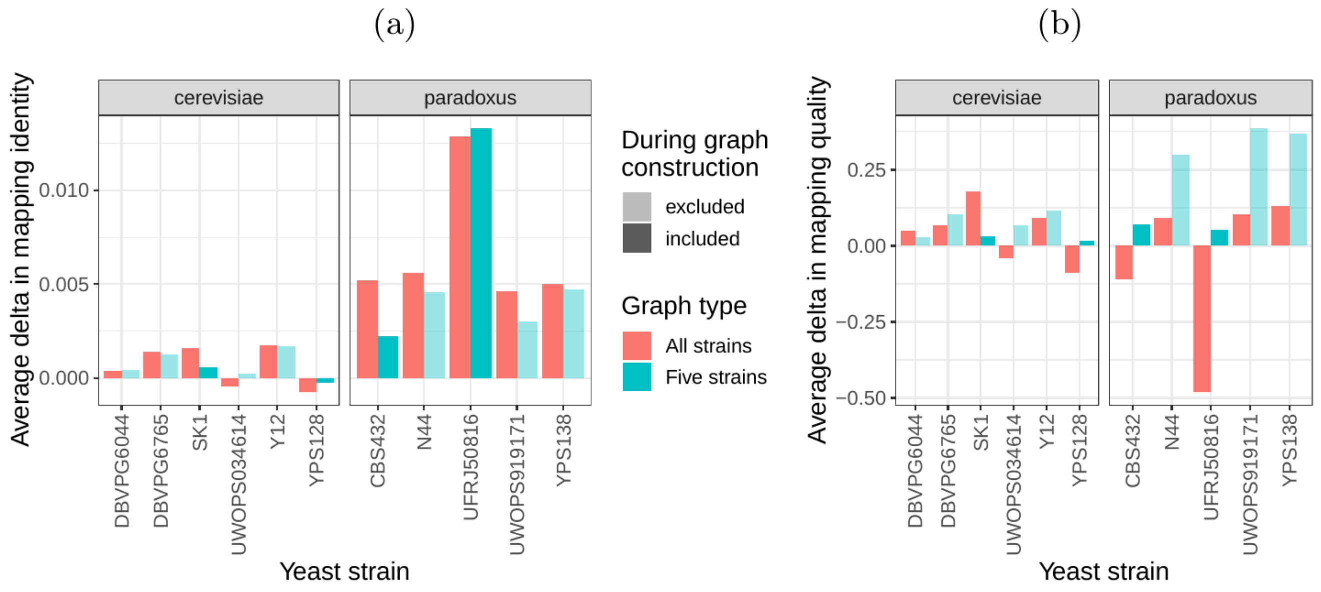

Fig. S15: SV genotyping comparison using all reads. Short reads from all 11 non-reference yeast strains were used to genotype SVs contained in the *cactus graph* and the *VCF graph*. Subsequently, sample graphs were generated from the resulting SV callsets. The short reads were aligned to the sample graphs and the quality of all alignments was used to ascertain SV genotyping performance. More accurate genotypes should result in sample graphs that have mappings with high identity and confidence for a greater proportion of the reads. a) Average delta in mapping identity of all short reads aligned to the sample graphs derived from *cactus graph* and *VCF graph*. b) Average delta in mapping quality of all short reads aligned to the sample graphs derived from *cactus graph* and *VCF graph*. Positive values denote an improvement of the *cactus graph* over the *VCF graph*. Colors represent the two strain sets and transparency indicates whether the respective strain was part of the *five strains set*.

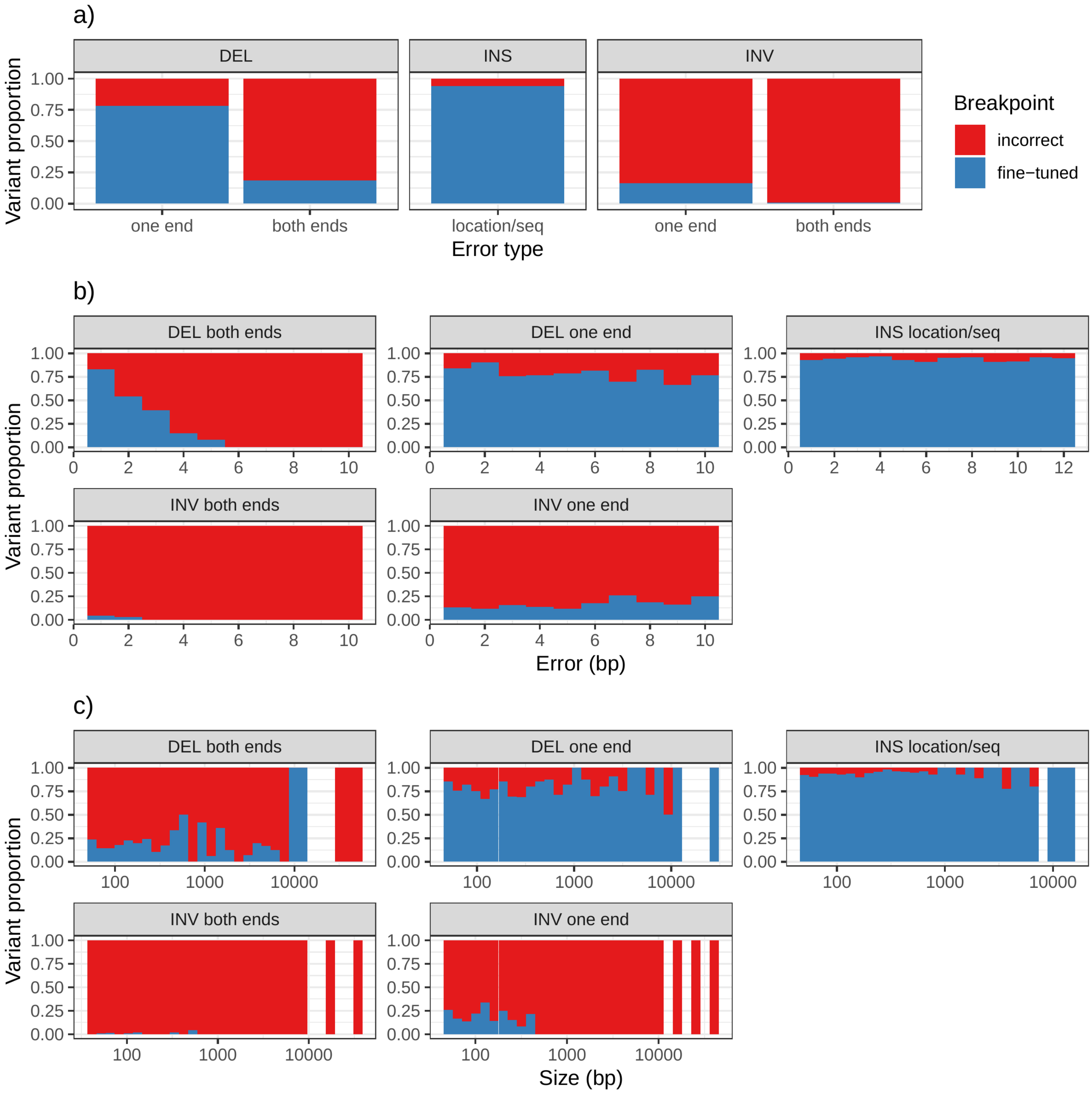

Fig. S16: Breakpoint fine-tuning using augmentation through "vg call". For deletions and inversions, either one or both breakpoints were shifted to introduce errors in the input VCF. For insertions, the insertion location and sequence contained errors. a) Proportion of variant for which breakpoints could be fine-tuned. b) Distribution of the amount of errors that could be corrected or not. c) Distribution of the size of the variants whose breakpoints could be fine-tuned or not.

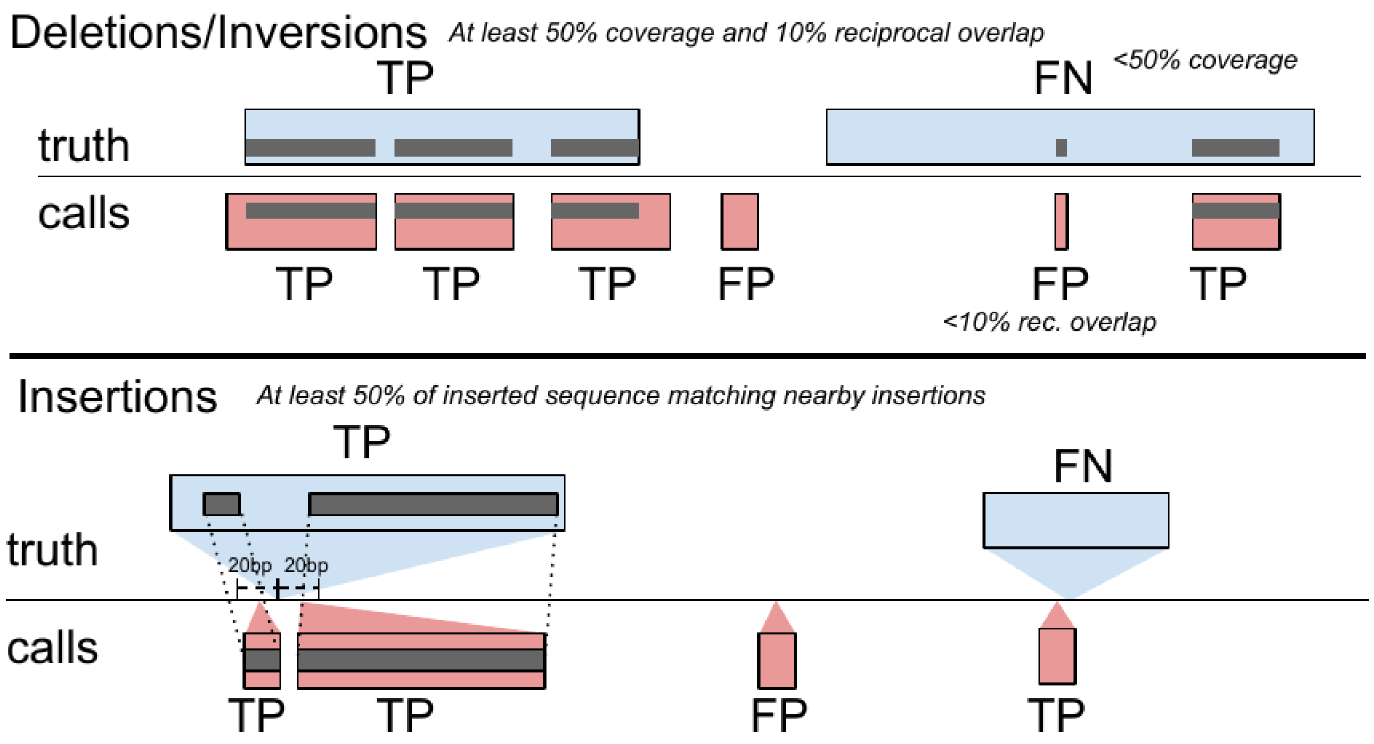

Fig. S17: Overview of the SV evaluation by the *sveval* package. For deletions and inversions, we compute the proportion of a variant that is covered by variants in the other set, considering only variants overlapping with at least 10% reciprocal overlap. A variant is considered true positive if this coverage proportion is higher than 50% and false-positive or false-negative otherwise. A similar approach is used for insertions, although they are first clustered into pairs located less than 20 bp from each other. Then their inserted sequences are aligned to derive the coverage statistics. The SV evaluation approach is described in more detail in the Methods.

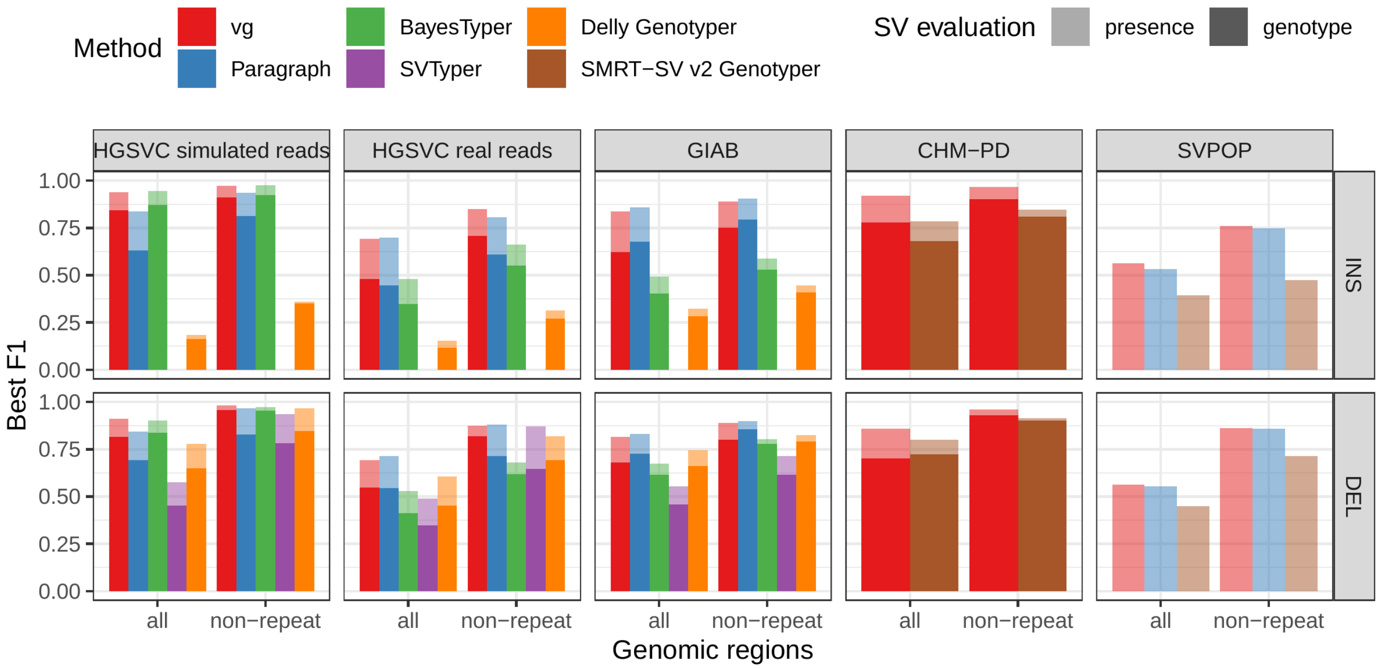

Fig. S18: Benchmark summary when using a more stringent matching criterion. At least 90% coverage was necessary to consider a variant matched, instead of the 50% minimum coverage used in other figures.

Supplementary Information

Variation graph and structural variation

A variation graph encodes DNA sequence in its nodes. Such graphs are bidirected, in that we distinguish between edges incident on the starts of nodes from those incident on their ends. A path in such a graph is an ordered list of nodes where each is associated with an orientation. If a path walks from, for example, node A in the forward orientation to node B in the reverse orientation, then an edge must exist from the end of node A to the end of node B. Concatenating the sequences on each node in the path, taking the reverse complement when the node is visited in reverse orientation, produces a DNA sequence. Accordingly, variation graphs are constructed so as to encode haplotype sequences as walks through the graph. Variation between sequences shows up as bubbles in the graph (5).

Breakpoint fine-tuning

In addition to genotyping, vg can use an augmentation step to modify the graph based on the read alignment and discover novel variants. On the simulated SVs, this approach was able to correct many of the 1-10 bp breakpoint errors that were added to the input VCF. The breakpoints were accurately fine-tuned for 93.8% of the insertions (Fig. S16a and Table S6). For deletions, 78.1% of the variants were corrected when only one breakpoint had an error. In situations where both breakpoints of the deletions were incorrect, only 18.6% were corrected through graph augmentation, and only when the amount of error was small (Fig. S16b). The breakpoints of less than 20% of the inversions could be corrected. Across all SV types, the size of the variant didn't affect the ability to fine-tune the breakpoints through graph augmentation (Fig. S16c).

Mappability comparison between yeast graphs

In order to elucidate whether the *cactus graph* represents the sequence diversity among the yeast strains better than the *VCF graph*, we mapped Illumina short reads to both graphs using `vg map`. Generally, more reads mapped to the *cactus graph* with high identity (Figs. S13a and S14a) and high mapping quality (Figs. S13b and S14b) than to the *VCF graph*. The *VCF graph* exhibited higher mappability only on the reference strain *S.c. S288C* with a marginal difference. The benefit of using the *cactus graph* is largest for strains in the *S. paradoxus* clade and smaller for strains in the *S. cerevisiae* clade. We found that the genetic distance to the reference strain (as estimated using Mash v2.1 (6)) correlated with the increase in confidently mapped reads (mapping quality >= 60) between the *cactus graph* and the *VCF graph* (Spearman's rank correlation, p-value=3.993e-06). These results suggest that the improvement in mappability is not driven by the higher sequence content in the *cactus graph* alone (16.8 / 15.4 Mb in the *cactus graph* compared to 12.6 / 12.4 Mb in the *VCF graph* for the *all strains set* and the *five strains set*, respectively). Instead, an explanation could be the construction of the *VCF graph* from a comprehensive but still limited list of variants and the lack of SNPs and small Indels in this list. Consequently, substantially fewer reads mapped to the *VCF graph* with perfect identity (Figs. S13a and S14a, percent identity threshold = 100%) than to the *cactus graph*. The *cactus graph* has the advantage of implicitly incorporating variants of all types and sizes from the *de novo* assemblies. As a consequence, the *cactus graph* captures the genetic makeup of each strain more comprehensively and enables more reads to be mapped.

Interestingly, our measurements for the *five strains set* showed only small differences between the five strains that were used to construct the graph and the other seven strains (Fig. S13). Only the number of alignments with perfect identity is substantially lower for the strains that were not included in the creation of the graphs (Fig. S13a).

Running time comparison between different tools for HG00514 as genotyped on the HGSVC dataset

SMRT-SV v2 Genotyper required roughly 36 hours and 30G ram on 30 cores to genotype the three HGSVC samples on the "SVPOP" VCF. These numbers are not directly comparable to the above table because 1) they apply to the "SVPOP" rather than "HGSVC" dataset (upon which we were unable to run SMRT-SV v2 Genotyper) and 2) we were unable to install SMRT-SV v2 Genotyper on AWS nodes and ran it on an older, shared server at UCSC instead.

Delly Genotyper, SVTyper and Paragraph start from a set of aligned reads, hence we also show the running time for read alignment with `bwa mem`(2).

For BayesTyper, the numbers include both khmer counting with kmc and genotyping. We note that BayesTyper integrated variant calls from GATK haplotypecaller(3) and Platypus(4), derived from reads mapped with bwa mem(2). The numbers shown for BayesTyper does not include this variant discovery pipeline.

Note: toil-vg reserves 200G memory by default for `vg snarls`. For this graph, about an order of magnitude less was required. It could have been run on 10 cores on 5 nodes instead.

References

1. Audano PA, Sulovari A, Graves-Lindsay TA, Cantsilieris S, Sorensen M, Welch AE, Dougherty ML, Nelson BJ, Shah A, Dutcher SK, Warren WC, Magrini V, McGrath SD, Li YI, Wilson RK, Eichler EE. Characterizing the Major Structural Variant Alleles of the Human Genome. Cell. 2019 Jan;176(3):663–675.e19. DOI: 10.1016/j.cell.2018.12.019

2. Heng Li. Aligning sequence reads, clone sequences and assembly contigs with BWA-MEM. arXiv. arXiv; 2013 Mar. Report No.: 1303.3997v2.

3. DePristo MA, Banks E, Poplin R, Garimella KV, Maguire JR, Hartl C, Philippakis AA, del Angel G, Rivas MA, Hanna M, McKenna A, Fennell TJ, Kernytsky AM, Sivachenko AY, Cibulskis K, Gabriel SB, Altshuler D, Daly MJ. A framework for variation discovery and genotyping using next-generation DNA sequencing data. Nat Genet. 2011 Apr 10;43(5):491–8. DOI: 10.1038/ng.806

4. Rimmer APhan H, Mathieson I, Iqbal Z, Twigg SRF, WGS500 Consortium, Wilkie AOM, McVean G, Lunter G. Integrating mapping-, assembly- and haplotype-based approaches for calling variants in clinical sequencing applications. Nat Genet. 2014 Jul 13;46(8):912–8. DOI: 10.1038/ng.3036

5. Paten B, Eizenga JM, Rosen YM, Novak AM, Garrison E, Hickey G. Superbubbles, Ultrabubbles, and Cacti. Journal of Computational Biology. 2018 Jul;25(7):649–63. DOI: 10.1089/cmb.2017.0251

6. Ondov BD, Treangen TJ, Melsted P, Mallonee AB, Bergman NH, Koren S, Phillippy AM. Mash: fast genome and metagenome distance estimation using MinHash. Genome Biol. 2016 Jun 20;17(1). DOI: 10.1186/s13059-016-0997-x
