# Supplementary material for: Genotyping structural variants in pangenome graphs using the vg toolkit
Source: Genome Biol. 2020 Feb 12;21:35. doi: 10.1186/s13059-020-1941-7 (PMC7017486; doi:10.1186/s13059-020-1941-7)
Supplement: Supplementary file 2 — Review history. [file 13059_2020_1941_MOESM2_ESM.docx]

# **1^st^ round**

**Reviewer 1**

This manuscript presents a method for genotyping structural variants using short reads in variation graphs, more specifically in the vg framework. This is a bioinformatics advance with important biological and clinical applications. The method, vg, appears to outperform (or in some case, perform nearly as well as) all other genotypers, including graph-based ones; especially for genotyping insertions, which are arguably harder to genotype than deletions.

My main concern is that the vg genotyping algorithm is not described in enough details, i.e. the "The vg call genotyping algorithm" section should be expanded to explain how read support is actually computed, to describe the genotyping statistical model (if there is any), how variants are projected into VCF, how the heuristic path search is performed (e.g. what are the cut-offs). There is also no mention of running time / memory usage, however the graph-alignment section clearly appears to have limited applicability due to performance considerations. I also have a number of other remarks detailed below.

Nevertheless, I found the manuscript to be sufficiently well written and the results to be important enough to consider that it should be conditionally accepted, depending on the quality of revisions. Also I would like to congratulate the authors for providing not just the open-source software but also many details for reproducibility.

Remarks:

1. In the abstract: " the vg toolkit now incorporates SVs in its unified variant calling framework": the right term in that sentence should probably not be 'variant calling' but 'genotyping', so as not to confuse that actual SV calling (i.e. the creation of SV catalog) is being performed. I noticed throughout the paper that the line between 'variant calling' and 'genotyping' is sometimes blurry. This should be clarified.

2. Feel free to mention in the introduction the Paragraph pre-print (https://www.biorxiv.org/content/biorxiv/early/2019/05/10/635011.full.pdf) even though it does not yet appear to have been reviewed, that work appears to be noteworthy.

3. Regarding the sentence "State-of-art methods like SVTyper[3] and Delly" is should be mentioned that Delly is not only a genotyper but also a caller.

4. Regarding the sentence "SMRT-SV v2 uses a different approach ..". It would be helpful to indicate (even if it is currently clear but implicit) that only the short-read genotyping module of SMRT-SV2 was used, not the main long-read variant calling module.

5. "Sites of variation are then identified using the snarl (aka "bubble") decomposition as described .." This sentence is not sufficient to get even a high-level intuition of what a snarl decomposition is.

6. "Generally, more reads mapped to the cactus graph with high identity (Figures S12a and S13a) and high mapping quality (Figures S12b and S13b) than to the VCF graph." Please add quantifications for both of these statements.

7. Section "Graphs from alignment of de novo assemblies" is hard to follow. The header and first paragraph of the section imply that graphs will be constructed from alignments, but the first graph presented is one constructed from a VCF file (VCF graph), not from an alignment.

8. Also, in the rest of this section, SV calling (not genotyping) is performed using vg. The two terms (calling and genotyping) appear to be used exchangeably in this paragraph, although they have different meanings.

9. In that section also, it would be helpful to give at least an intuition of what the 'sample graph' is, until it is properly defined in the methods.

10. Finally, also in the "Graphs from alignment of de novo assemblies" section, genotyping results are of different nature than the previous section; i.e. no F1 score is shown. Why couldn't true positives/true negatives/etc be evaluated in that setting as well?

11. No indication of the time/memory taken for the analyses appears in the main text. Could you please give an indication of the performance of the software?

12. It would be appropriate to give the current upper limit in terms of genome size for creating graphs from alignments; in particular, are yeast-sized genomes the limit?

13. In the methods, the transition between the 3-point description of the vg calling algorithm and 'toil-vg' is a bit rough. Please explain earlier what the "toil-*" sections are overall going to be about.

14. In the methods, section "The vg call genotyping algorithm", how is the genotype determined in step 3?

15. In the methods, the citation for kmc is in url form, please replace it by a proper citation.

**Reviewer 2**

The authors present new work to extend the vg tool set to call and genotype structural variants (defined as polymorphisms affecting more than 50bp of sequence). The vg software is collection of algorithms for representing multiple sequenced haplotypes in a variation graph structure. The extensions to the vg tools to handle structural variation are described as are the results of benchmarking studies to compare vg to other existing tools for calling and genotyping structural variation. The authors also use a collection of data on 12 yeast strains to evaluate the benefits of building variation graphs from de novo assemblies vs. catalogs of previously ascertained variants.

I have two major comments and some more minor comments.

Major Comments

First, the metrics used to determine genome accuracy are based not on comparing the genotype calls for a set of identical alleles (as would be done for SNPs), but instead a more complex matching procedure is used where a variant in one genotyping call set must be "sufficiently covered" by variants in another genotyping call set (on the same sample) to be counted as a matching or non-matching genotype. This evaluation procedure is described in detail in the methods section and the code implementing it is available online.

I understand the motivation to do this, which is that often the allelic representations created by different tools may differ. I do wonder, though, whether this is exacerbated by the use of a variant graph, where small bubbles in the graph could be construed as different alleles or whether many similar (but theoretically distinct) paths through the graph could all be considered as distinct alleles.

One specific recommendation is to make it more clear to the casual reader how genotyping accuracy is being measured and as part of this perhaps include a brief discussion of the difficulties in comparing genotypes for structural variants and thus why you have adopted this methodology.

I would also welcome some additional data on how much (or how little) the differences in allelic representation affect the overall conclusions in the paper. For example, if two genotypes "match" according to the author's criteria, then how different are these alleles most of the time or on the tail of the "matching" distribution? How many times does a "match" occur where the called variants are not one-to-one but are instead one-to-many or many-to-many? How sensitive are the results to particular thresholds (for example, the use of 50% reciprocal overlap)?

My second major comment is that there is no discussion of computational performance. As the authors describe, there is a huge need to be able to scale up these kinds of analyses to large studies involving tens or hundreds of thousands of genomes. It would be great to have some sense of the cost and challenges involved in performing graph-based analyses at scale today and how this differs between the other methods benchmarked.

Minor Comments

Line 46: "state-of-the-art"

Line 77: They authors list 3 existing "graph-based" methods: GraphTyper, BayesTyper and SMART-SV v2. Of these, they do not do a comparative analysis to GraphTyper. I think this omission deserves at least more explanation.

Line 111: "in detail"

Line 115: The x-axis should also be explained in the legend. My understanding is that it is the simulated read depth.

Line 124: Actually, the data presented do not look like a tie to me between Delly and vg for deletions. Delly seems to be marginally better at all depths (given accurate breakpoints). My suggestion would be to reduce the amount of detailed comparison in this section and instead focus on the point in the last sentence of this section: This section is framed at the outset as a proof-of-concept and as such the results shows that the vg approach is at least competitive with existing methods.

Line 266: Although this is explained earlier in the text, I feel like it might be good to make sure this sentence cannot be taken out of context: Specifically, I would recommend saying "The mean improvements in mapping identity across the strains (for reads that differed in mapping identity), was ...". Otherwise, I worry a reader might interpret these numbers as being derived from the all-reads experiment (figure S14).

Line 287: I found this first sentence a little off-putting: "Overall, vg was the most accurate sv genotyper in our benchmarks." My reaction has I think more to do with tone than with absolute truth. I feel like the analyses showed that vg is quite good, generally better, but not overwhelmingly better. An important conclusion (to me) is that graph-based methods (in general) show evidence of out-performing the other methods that were compared. Overall, the accuracy of all of the methods still leaves a lot to be desired and I would hope that future improvements could be made in all of the graph-based methods.

Line 302: I was surprised that the discussion of breakpoint correction/fine-tuning was not included until near the end in the discussion section. I found this of great interest, and I think the paper would be improved by highlighting this work more and perhaps by extending it to see how well it works with real data instead of just simulated reads. Something that I think would be really interesting is if you have encountered any cases where humans (or yeast) have similar but distinct alleles and vg can automatically and accurately disambiguate them across individuals even if the initial variation graph contains just one of the two alleles. This seems like a particular strength of vg compared to existing methods.

Related to this, I would suggest that the more interesting thing to plot in S14-c would be the proportion of correctable breakpoints (rather than the absolute number) stratified by length.

Overall, I found this work of great interest. If the authors address my comments above, I would recommend it for publication.

**Reviewer 3**

- Are the methods appropriate to the aims of the study, are they well described, and are necessary controls included?

Yes. The Major comments are:

1. Show runtime analysis for graph construction versus linear vcf, and genotyping of methods.

2. Show genotyping accuracy by SV type: mobile element insertion, VNTR, and other.

- Are the conclusions adequately supported by the data shown?

Yes.

- Are sufficient details provided to allow replication and comparison with related analyses that may have been performed?

Yes.

- Is the method likely to be of broad utility? Is any software component easy to install and use? Please indicate briefly the novel features and/or advantages of the method, and/or please reference the relevant publications and which methods, if any, it should be compared with.

Yes. The vg software is under rapid development.

- Is the paper of broad interest to others in the field, or of outstanding interest to a broad audience of biologists?

Yes.

# **Response to Reviewers**

## **Reviewer #1:**

This manuscript presents a method for genotyping structural variants using short reads in variation graphs, more specifically in the vg framework. This is a bioinformatics advance with important biological and clinical applications. The method, vg, appears to outperform (or in some case, perform nearly as well as) all other genotypers, including graph-based ones; especially for genotyping insertions, which are arguably harder to genotype than deletions.

My main concern is that the vg genotyping algorithm is not described in enough details, i.e. the "The vg call genotyping algorithm" section should be expanded to explain how read support is actually computed, to describe the genotyping statistical model (if there is any), how variants are projected into VCF, how the heuristic path search is performed (e.g. what are the cut-offs). There is also no mention of running time / memory usage, however the graph-alignment section clearly appears to have limited applicability due to performance considerations. I also have a number of other remarks detailed below.

Nevertheless, I found the manuscript to be sufficiently well written and the results to be important enough to consider that it should be conditionally accepted, depending on the quality of revisions. Also I would like to congratulate the authors for providing not just the open-source software but also many details for reproducibility.

**Remarks:**

1. In the abstract: " the vg toolkit now incorporates SVs in its unified variant calling framework": the right term in that sentence should probably not be 'variant calling' but 'genotyping', so as not to confuse that actual SV calling (i.e. the creation of SV catalog) is being performed. I noticed throughout the paper that the line between 'variant calling' and 'genotyping' is sometimes blurry. This should be clarified.

We have corrected the abstract and the text, replacing “calling” with “genotyping” (see manuscript with highlighted track changes).

2. Feel free to mention in the introduction the Paragraph pre-print (https://www.biorxiv.org/content/biorxiv/early/2019/05/10/635011.full.pdf) even though it does not yet appear to have been reviewed, that work appears to be noteworthy.

We have added a sentence in the introduction mentioning Paragraph.

*“Paragraph builds a graph for each breakpoint of known variants [20], then, for each breakpoint, it pulls out all nearby reads from the linear alignment and re-aligns them to the graph. Genotypes are computed using the read coverage from the pair of breakpoint graphs corresponding to each SV.”*

We have also integrated this method in our benchmark even if it is not yet published.

3. Regarding the sentence "State-of-art methods like SVTyper[3] and Delly" is should be mentioned that Delly is not only a genotyper but also a caller.

This sentence now reads: “*Current methods such as SVTyper[3] and the genotyping module of Delly[4] (henceforth referred to as Delly Genotyper) typically focus on split reads and paired reads mapped too close or too far from each other*.”

In the rest of the manuscript, we use “Delly Genotyper” to make it clear that we only use the genotyping module of Delly.

4. Regarding the sentence "SMRT-SV v2 uses a different approach ..". It would be helpful to indicate (even if it is currently clear but implicit) that only the short-read genotyping module of SMRT-SV2 was used, not the main long-read variant calling module.

We now refer to this method as “SMRT-SV2 Genotyper” to make this distinction more clear.

5. "Sites of variation are then identified using the snarl (aka "bubble") decomposition as described .." This sentence is not sufficient to get even a high-level intuition of what a snarl decomposition is.

A few sentences were added here, and the Methods section was expanded considerably to address your concern above:

*“Sites of variation within the graph are then identified using the snarl decomposition as described in [24]. These sites correspond to intervals along the reference paths (ex. contigs or chromosomes) which are embedded in the graph. They also contain nodes and edges deviating from the reference path, which represent variation at the site. For each site, the two most supported paths between its interval (haplotypes) are determined, and their relative supports used to produce a genotype at that site (Figure 1a).”*

6. "Generally, more reads mapped to the cactus graph with high identity (Figures S12a and S13a) and high mapping quality (Figures S12b and S13b) than to the VCF graph." Please add quantifications for both of these statements.

Following this suggestion, two sentences were added:

*“On average, 88%, 79%, and 68% of reads mapped to the all strain cactus graph with an identity of at least 50%, 90%, and 100%, respectively, compared to only 77%, 57%, and 23% of reads on the all strain VCF graph. Similarly, 88% of reads mapped to the all strain cactus graph with a mapping quality of at least 30 compared to only 80% of reads on the all strain VCF graph”.*

7. Section "Graphs from alignment of de novo assemblies" is hard to follow. The header and first paragraph of the section imply that graphs will be constructed from alignments, but the first graph presented is one constructed from a VCF file (VCF graph), not from an alignment.

Thank you for this comment. The second paragraph of the section did not make it clear that the “VCF graph” is used here as a baseline to compare against the alignment-derived “cactus graph”. The “VCF graph” represents the graph construction approach that is used for the human experiments throughout the manuscript. In contrast to that approach which relies on existing SV catalogs in VCF format, the “cactus graph” is generated directly from genome-genome alignments. We re-phrased the second paragraph of the “Graphs from alignment of de novo assemblies” section to make this clearer, including the new sentence:

*“We compared genotyping results from a WGA-derived graph (cactus graph) with results from a VCF-derived graph (VCF graph).”*

8. Also, in the rest of this section, SV calling (not genotyping) is performed using vg. The two terms (calling and genotyping) appear to be used exchangeably in this paragraph, although they have different meanings.

Yes, this can indeed cause confusion because the distinction between variant calling and genotyping can become somewhat blurry for alignment-derived graphs. When several genome assemblies (including one reference genome) are aligned with the tool Cactus, a genome graph with an embedded reference path is generated. Nodes and edges that are not part of the reference path represent variants. After mapping reads to the graph, toil-vg call traverses the graph and the embedded variant sites to output genotype calls. It is a rather philosophical question whether toil-vg call performs variant calling and genotyping simultaneously using the genome graph. Another interpretation could be that the variant calling implicitly happens during graph construction with Cactus and that toil-vg call only performs genotyping.

Because our pipeline produces genotypes we updated the manuscript to reflect that we perform genotyping and not only variant calling. In the text “SV calling” was replaced with “SV genotyping”, “called variants” with “genotyped variants”, and “callset” with “genotype set”.

9. In that section also, it would be helpful to give at least an intuition of what the 'sample graph' is, until it is properly defined in the methods.

Following the reviewer’s suggestion, we added the following sentence:

*Conceptually, the sample graph represents the sample’s diploid genome by starting out from the reference genome and augmenting it with the genotype results.*

10. Finally, also in the "Graphs from alignment of de novo assemblies" section, genotyping results are of different nature than the previous section; i.e. no F1 score is shown. Why couldn't true positives/true negatives/etc be evaluated in that setting as well?

The genotyping results are different because of the lack of a gold standard for the yeast strains. In order to compute measures, such as recall, precision, and F1 score, a comparison set of “known” genotypes is needed to determine true positives, false positives, and false negatives. Due to the lack of such a comparison set, we evaluated the genotyping results with the indirect approach of mapping reads to the sample graphs. To make this more explicit, we extended the following sentence:

*“There is no gold standard available for these samples to compare against which renders the evaluation of recall, precision and F1 score impossible.”*

11. No indication of the time/memory taken for the analyses appears in the main text. Could you please give an indication of the performance of the software?

This is an important point that was indeed not included in the manuscript. We added a Table S7 with time and memory estimates for vg and the other benchmarked methods.

12. It would be appropriate to give the current upper limit in terms of genome size for creating graphs from alignments; in particular, are yeast-sized genomes the limit?

The methods to align whole-genomes and to manipulate variation graphs (with vg) can already deal with multi-gigabase genomes. However, for larger and more complex genomes, we will need to do more work investigating the quality of these alignments and the performance of read mapping and variant genotyping. We believe the approach that we used on yeast could already be used on larger genomes. We have been gathering a number of high-quality de novo human assemblies and as mentioned in the Discussion, we are working now on applying this approach to human genomes.

13. In the methods, the transition between the 3-point description of the vg calling algorithm and 'toil-vg' is a bit rough. Please explain earlier what the "toil-*" sections are overall going to be about.

We thank the reviewer for this suggestion. This section has been re-worked. The genotyping algorithm is explained first, in detail, then toil-vg is touched on briefly.

14. In the methods, section "The vg call genotyping algorithm", how is the genotype determined in step 3?

This section has been rewritten to give more details, including the exact thresholds to predict genotypes.

15. In the methods, the citation for kmc is in url form, please replace it by a proper citation.

We thank the reviewer for letting us know about the oversight. The reference is now cited properly.

## **Reviewer #2**

The authors present new work to extend the vg tool set to call and genotype structural variants (defined as polymorphisms affecting more than 50bp of sequence). The vg software is collection of algorithms for representing multiple sequenced haplotypes in a variation graph structure. The extensions to the vg tools to handle structural variation are described as are the results of benchmarking studies to compare vg to other existing tools for calling and genotyping structural variation. The authors also use a collection of data on 12 yeast strains to evaluate the benefits of building variation graphs from de novo assemblies vs. catalogs of previously ascertained variants.

I have two major comments and some more minor comments.

**Major Comments:**

First, the metrics used to determine genome accuracy are based not on comparing the genotype calls for a set of identical alleles (as would be done for SNPs), but instead a more complex matching procedure is used where a variant in one genotyping call set must be "sufficiently covered" by variants in another genotyping call set (on the same sample) to be counted as a matching or non-matching genotype. This evaluation procedure is described in detail in the methods section and the code implementing it is available online.

I understand the motivation to do this, which is that often the allelic representations created by different tools may differ. I do wonder, though, whether this is exacerbated by the use of a variant graph, where small bubbles in the graph could be construed as different alleles or whether many similar (but theoretically distinct) paths through the graph could all be considered as distinct alleles.

One specific recommendation is to make it more clear to the casual reader how genotyping accuracy is being measured and as part of this perhaps include a brief discussion of the difficulties in comparing genotypes for structural variants and thus why you have adopted this methodology.

I would also welcome some additional data on how much (or how little) the differences in allelic representation affect the overall conclusions in the paper. For example, if two genotypes "match" according to the author's criteria, then how different are these alleles most of the time or on the tail of the "matching" distribution? How many times does a "match" occur where the called variants are not one-to-one but are instead one-to-many or many-to-many? How sensitive are the results to particular thresholds (for example, the use of 50% reciprocal overlap)?

We thank the reviewer for this detailed comment. We have added the rationale for using our evaluation approach at the beginning of the relevant method section:

*“toil-vg sveval evaluates the SV calls relative to a truth set. Matching SV calls is non-trivial because two SV callsets often differ slightly around the breakpoints. Even for a genotyping experiment, the same input SVs can have equivalent but different representations. Furthermore, SV catalogs often contains very similar SVs that could be potentially duplicates of the same true variant. To make sure that SVs are matched properly when comparing genotyped SVs and the truth set, we use an approach that overlaps variants and aligns allelic sequences if necessary. It was implemented in the sveval R package (https://github.com/jmonlong/sveval). Figure S17 shows an overview of the SV evaluation approach which is described below. Of note, the variants are first normalized with bcftools norm (1.9) to ensure consistent representation between called variants and baseline variants[36].”*

We have rerun the full evaluation using a more stringent coverage threshold of 90% instead of 50%. These results, now in Figure S18, show a slight decrease in terms of accuracy. The marginal drop suggests that most genotyped and true variants overlap significantly.

Furthermore, we have explored the amount of matched variants in the genotyped set versus the truth set. This allowed us to estimate how many variants are matched to multiple others. We have added a supplementary Figure S5 and discuss this analysis in the Results section. Interestingly this highlighted some limitations for tools that genotype each variant independently. The new paragraph in the Results section read:

*“In general, the genotyped variants were matched 1-to-1 with variants in the truth set but some methods showed some signs of “over-genotyping” that is not reflected in the precision/recall/F1 scores. Methods like Paragraph, Delly Genotyper or SVTyper tended to genotype on average more than one variant per truth-set variant (Figure S5). Like other SV catalogs, the HGSVC catalog is not fully sequence-resolved and contains a number of near-duplicates with slightly different breakpoint definition. When genotyping a sample, multiple versions of a variant are genotyped multiple times by methods that analyze each variant independently. In contrast, vg follows a unified path-centric approach that only select the best genotype in a region (see Methods).”*

My second major comment is that there is no discussion of computational performance. As the authors describe, there is a huge need to be able to scale up these kinds of analyses to large studies involving tens or hundreds of thousands of genomes. It would be great to have some sense of the cost and challenges involved in performing graph-based analyses at scale today and how this differs between the other methods benchmarked.

This is an important point that was indeed not included in the manuscript. We added a Table S7 with time and memory estimates for vg and the other benchmarked methods.

**Minor Comments**

Line 46: "state-of-the-art"

This comment has been addressed.

Line 77: They authors list 3 existing "graph-based" methods: GraphTyper, BayesTyper and SMART-SV v2. Of these, they do not do a comparative analysis to GraphTyper. I think this omission deserves at least more explanation.

GraphTyper was not included in the benchmark because it can only genotype SNVs and short indels. We have left the sentence written as:

*“GraphTyper realigns mapped reads to a graph built from known SNVs and short indels using a sliding-window approach[18].”*

Line 111: "in detail"

The legend as been corrected with the suggested form.

Line 115: The x-axis should also be explained in the legend. My understanding is that it is the simulated read depth.

It is indeed the simulated read depth. The caption has been updated to include:

*“For each experiment, the x-axis is the simulated read depth and the y-axis shows the maximum F1 across different minimum quality thresholds.”*

Line 124: Actually, the data presented do not look like a tie to me between Delly and vg for deletions. Delly seems to be marginally better at all depths (given accurate breakpoints). My suggestion would be to reduce the amount of detailed comparison in this section and instead focus on the point in the last sentence of this section: This section is framed at the outset as a proof-of-concept and as such the results shows that the vg approach is at least competitive with existing methods.

The text in this paragraph changed substantially, mostly because of the addition of Paragraph in the benchmark. The claim that Delly and vg tied for deletion is not present anymore. Following the reviewer’s suggestion we have also reduced the details in this paragraph to focus on the main result of this section, that vg is competitive with existing approaches in simple proof-of-concept simulations.

Line 266: Although this is explained earlier in the text, I feel like it might be good to make sure this sentence cannot be taken out of context: Specifically, I would recommend saying "The mean improvements in mapping identity across the strains (for reads that differed in mapping identity), was ...". Otherwise, I worry a reader might interpret these numbers as being derived from the all-reads experiment (figure S14).

This is a good point. We incorporated this suggested change into the manuscript.

Line 287: I found this first sentence a little off-putting: "Overall, vg was the most accurate sv genotyper in our benchmarks." My reaction has I think more to do with tone than with absolute truth. I feel like the analyses showed that vg is quite good, generally better, but not overwhelmingly better. An important conclusion (to me) is that graph-based methods (in general) show evidence of out-performing the other methods that were compared. Overall, the accuracy of all of the methods still leaves a lot to be desired and I would hope that future improvements could be made in all of the graph-based methods.

The reviewer makes a good point. We have changed the tone of this paragraph which now starts with:

*“Overall, graph-based methods were more accurate than traditional SV genotypers in our benchmarks, with vg performing best across most datasets.”*

Line 302: I was surprised that the discussion of breakpoint correction/fine-tuning was not included until near the end in the discussion section. I found this of great interest, and I think the paper would be improved by highlighting this work more and perhaps by extending it to see how well it works with real data instead of just simulated reads. Something that I think would be really interesting is if you have encountered any cases where humans (or yeast) have similar but distinct alleles and vg can automatically and accurately disambiguate them across individuals even if the initial variation graph contains just one of the two alleles. This seems like a particular strength of vg compared to existing methods.

We share the reviewer’s enthusiasm about vg’s potential for breakpoint fine-tuning. We had planned to work on correcting SV catalogs using graph augmentation. As the reviewer suggested, it might allow us to remove potential duplicates in the catalogs and fine-tune SV breakpoints. However this represents a substantial amount of work as we would like to use a large cohort of genomes for this analysis. For this reason, we only mention our preliminary results in the discussion.

Related to this, I would suggest that the more interesting thing to plot in S14-c would be the proportion of correctable breakpoints (rather than the absolute number) stratified by length.

We thank the reviewer for this suggestion. The proportion of variant is indeed more relevant and is now shown in sub-figures b) and c).

Overall, I found this work of great interest. If the authors address my comments above, I would recommend it for publication.

## **Reviewer #3**

- Are the methods appropriate to the aims of the study, are they well described, and are necessary controls included?

Yes. The Major comments are:

1. Show runtime analysis for graph construction versus linear vcf, and genotyping of methods.

This is an important point that was indeed not included in the manuscript. We added a Table S7 with time and memory estimates for vg and the other benchmarked methods.

2. Show genotyping accuracy by SV type: mobile element insertion, VNTR, and other.

We have performed a new analysis to annotate the deleted/inserted sequence with RepeatMasker in order to provide genotyping estimates for different classes of transposable element polymorphisms and repeats. The results are shown in a new Figure S6 and the Results now include the following sentences:

*“By annotating the repeat content of the deleted/inserted sequence we further evaluated vg’s performance across repeat classes. As expected, simple repeat variation was more challenging to genotype than transposable element polymorphisms (Figure S6).”*

- Are the conclusions adequately supported by the data shown?

Yes.

- Are sufficient details provided to allow replication and comparison with related analyses that may have been performed?

Yes.

- Is the method likely to be of broad utility? Is any software component easy to install and use? Please indicate briefly the novel features and/or advantages of the method, and/or please reference the relevant publications and which methods, if any, it should be compared with.

Yes. The vg software is under rapid development.

- Is the paper of broad interest to others in the field, or of outstanding interest to a broad audience of biologists?

Yes.

**2nd Round**

**Reviewer 1**

The authors have provided satisfactory responses to my comments.

In particular, a software (Paragraph) from a recent preprint was incorporated into the benchmarks, even though I did not explicitly request that change -- had only suggested to mention it. This addition is commendable as the good performance of Paragraph puts vg into a less favorable light in terms of novelty. But this adds to the completeness of the paper and overall reflects the scientific integrity of the authors.

I went through the revised methods section and have no further comments.

**Reviewer 2**

The authors addressed all of my comments and concerns.

I commend the authors for evaluating Paragraph and including benchmarking results in the revised manuscript. It is interesting to see that the genotyping accuracy of Paragraph, which uses similar methods to vg, has similar genotyping accuracy.

I appreciate that the authors included more detail in the supplementary material in the revised draft and especially a more detailed description of the genotyping algorithm.

I note that Figure 3 (an example of one genotyped site) seems to be missing in the revised submission, or at least I didn't see it, but I presume it is unchanged from the original manuscript.
